# Sustainable rural housing in cold climates: a model for Rumicruz-Ecuador

**DOI:** 10.12688/f1000research.162591.2

**Published:** 2025-07-29

**Authors:** Karina Elizabeth Cajamarca Dacto, Jean Carlos Montero Riofrio, Jorge Luis Gallegos Rodríguez, Israel Fernando Noriega Cadena

**Affiliations:** 1 Facultad de Ingeniería, Universidad Nacional de Chimborazo, Riobamba, Chimborazo Province, 060110, Ecuador; 2 independent researcher, Riobamba, Chimborazo, 060110, Ecuador

**Keywords:** Vernacular, thermal comfort, housing, sustainability, design, bioclimatic, Rumicruz

## Abstract

**Background:**

The loss of cultural identity in the rural architecture of Rumicruz, Chimborazo, is a consequence of the adoption of generic models and unsustainable modern materials, which has affected thermal comfort and the connection with local traditions. This research proposes sustainable housing that combines vernacular and modern techniques, respecting both the environment and the community’s needs.

**Method:**

Based on a mixed approach (qualitative and quantitative), the architectural, social, and environmental context was analyzed through a field diagnosis, literature review and critical observation. During this process, problems related to thermal comfort, housing deterioration, and the use of inadequate materials were identified. The proposal includes the use of mudbrick, stone, and concrete blocks, complemented with solar heating systems and energy efficiency, significantly improving thermal comfort compared to traditional housing.

**Conclusions:**

It is concluded that the proposal integrates cultural identity and sustainability, adapting traditional techniques to current demands. The bioclimatic analysis and design support the thermal comfort values, highlighting the relevance of designs adapted to the local context.

## 1. Introduction

Understanding that rural housing should be a way to improve the economy of the family, that is, it should be adapted to the conditions of the sector and to the new realities, understanding the ways of life and the socio-cultural constructions within a productive environment. ^1^

The lack of a tangible connection between architecture and cultural identity is reflected in the increasing uniformity of the architectural landscape. The indiscriminate use of modern materials has contributed to the loss of the diversity of traditional building styles and techniques, once a manifestation of local history. Dwelling, which once told the stories of generations, have been transformed into generic structures without a cultural narrative, depriving rural communities of an essential part of their architectural heritage. This phenomenom causes a disconnection between current and previous generations, devaluing traditional building practices and putting at risk a heritage that has evolved in symbiosis with rurality.

It is fundamental to consider that this symbiosis manages bioclimatic, sustainable, and ecological strategies that should be rescued, as well as the traditional architectural forms, textures, and native colors, based on local materials and pigments that reflect the cultural identity of the region. In addition, the anthropomorphic and functional aspect is emphasized, where spaces are adjusted to local human needs and scales according to their function. The use of traditional and renewable materials is also highlighted, along with the inherited construction methods, adapted to the local climate and respectful of the environment.

In the current context, throughout history, rural communities have been able to create housing solutions that not only meet the needs of their inhabitants, but also respect the natural environment and reflect their culture. However, in recent decades, the massive adoption of construction models alien to these contexts has generated a gap between traditional rural architecture and the demands of modern development. Rural housing can be an engine for sustainable development, preserving its cultural and ecological richness.

In this context, it is necessary to understand separately what rural housing refers to. Housing should be understood as a place that provides shelter, where the user experiences a sense of peace and appropriation of the place. On the other hand, rural housing is related to the closeness to nature and remoteness to urban areas, where the natural conditions of the environment can be better used to meet the needs of the users. ^2^

Rural housing is located in mountainous areas, far from urban zones, in agricultural sectors, whose land where the houses are built is inherited from parents to children. The houses do not have a technical direction and are born of spontaneity. ^3^ This type of architecture arises from the basic needs of the people and the place, being these characteristics the ones that allow it to be an architecture representative of the history and idiosyncrasy of its people, having by itself a natural feature that is evident in its composition and its growth, responding to their productive needs, as well as to the climate of the area. ^4^

Rural housing is defined by the building, the land used for productive activities, and the relationship with the surrounding environment (production and nearby properties). This rural housing is generally dispersed throughout the property, while in areas of greater consolidation, it is concentrated. In addition, it must comply with two fundamental qualities: being productive and sustainable. ^5^

The challenge of modernization in architecture seeks to generate a model of Sustainable Rural Housing based on economic balance, social inclusion, and environmental protection. To this end, it is crucial to rescue and adapt traditional construction techniques to current demands, using local and renewable materials that reduce the carbon footprint and promote the local economy. It is also essential to apply bioclimatic design principles to optimize energy use and adapt buildings to the scale and customs of the communities, prioritizing functionality without resorting to generic solutions. In this way, rural identity can be preserved and progress can be made towards a sustainable model.

Considering that the main objective of sustainable housing is to reduce the environmental impact of buildings by reducing the consumption of resources and energy efficiency, ^6^ it does not directly mean to reduce the quality of a service, but to use it in a more practical and sustainable way. ^7^ For this, local techniques and materials that avoid pollution and promote employment in the region are used. Therefore, sustainable housing should create efficient communities in the use of energy, water, soil, materials, and labor. Additionally, these dwellings should be designed to have a long useful life, be flexible to the needs of their users, healthy and recyclable. ^8^

It is evident that rural architecture has historically been a direct response to the local environment. The houses built in these areas not only serve to protect their inhabitants, but have been a clear example of adaptation to natural elements such as temperature, winds, solar radiation and humidity. This statement on rural architecture and its relationship with energy efficiency is supported by studies on traditional architecture. In these constructions, local materials such as limestone, mudbrick and straw are used, which offer thermal insulation and temperature regulation properties. This approach allows the buildings to adapt to extreme climatic conditions, keeping the interiors cool in summer and warm in winter. These techniques are also associated with circular economy and sustainability. ^9^

It is imperative to analyze how traditional buildings can inspire new ways to reduce dependence on artificial air conditioning systems. ^10^ In which the perception of well-being and satisfaction experienced by the users is estimated when they establish a certain permanence in a particular environment without the need to keep moving to maintain normal levels of body temperature. ^11^ It is also important to take into account that the conditions of thermal comfort will depend on natural factors such as temperature, wind, humidity, among others, as well as factors such as the activity that takes place in the place, clothing. Therefore, in the bioclimatic design of rural architecture it is crucial to consider bioclimatic strategies, properly selecting the shape of the house, its orientation, the distribution of spaces according to the user’s activities and the correct use of materials, to adapt to the climate of the site. ^12^ Thus, these vernacular construction techniques use local materials such as mudbrick, wood, straw, stone and other materials, generating a natural control of temperature and ventilation, through passive bioclimatic strategies, thus becoming an early model of sustainability.

However, the indiscriminate use of industrial materials such as concrete and steel, as well as the importation of construction styles from urban areas or from other countries, has contributed to the disappearance of these vernacular techniques and designs. This transformation not only affects the ecological efficiency of rural housing but also erodes the cultural identity of the communities. This global situation is not foreign to the province of Chimborazo, where the generalized belief that vernacular architecture is primitive or antiquated has directly contributed to the abandonment of these practices in favor of more modern methods, which are not necessarily more effective or sustainable.

The progressive loss of vernacular architecture is undoubtedly linked to several factors, among them the displacement of the rural population to the cities. This phenomenon has led to a transformation in construction methods, often resulting in the adoption of materials and techniques foreign to the local context. The migration of people from rural to urban areas not only changes the physical landscape but also the link with building traditions that have been passed down from generation to generation.

Therefore, it is worth noting that vernacular architecture, characterized by using local materials and techniques adapted to the climate and environment, offers numerous advantages: sustainability, energy efficiency, connection with local culture and identity, and a minimal environmental footprint. However, the advance of industrialization and the globalization of construction techniques has relegated many of these practices to the background, despite their historical and cultural relevance.

This change also responds to a perceived need for modernization, which seeks to imitate foreign architectural styles, without valuing the ancestral knowledge found in vernacular constructions. The loss of this wisdom not only affects the architecture but also diminishes the sense of belonging and cultural continuity in rural communities.

In the case of Rumicruz community, located in the parish of Calpi, province of Chimborazo, these social and territorial transformations dynamize the population as well as the rural and urban territory. As a result, the indigenous people leave aside their traditional activities to carry out internal, regional, and international migratory projects. ^13^

According to the detailed observation carried out in the community of Rumicruz, it has been possible to determine that vernacular housing has been losing continuity and being progressively replaced since the 1980s. That is to say, approximately four decades have passed since 1985, when houses with construction models foreign to rurality began to appear. These new buildings incorporate modern materials such as concrete, glass and steel in different elements, such as the structure, windows, doors and walls.

The local situation increases when the number of existing Andean vernacular dwellings disappears over the years. Currently, in this study area, only six vernacular dwellings that present these outstanding cultural and architectural features are preserved. Unfortunately, they have already lost their use as dwellings, becoming animal shelters or vestiges of what once were houses. ^14^

Of the six Andean vernacular dwellings still preserved in the community, the oldest dates from 1949 and the most recent from 1960. All of these dwellings have a single-story rural typology, isolated on the property, without windows, with adobe load-bearing walls and sloping thatched and reed roofs. In other words, they are built with local materials and respond to local climatic conditions.

However, from 1985 to the present, the houses in Rumicruz have undergone significant transformations in terms of their form, function, materials and typology, showing a progressive abandonment of their relationship with their surroundings and with the characteristics of rural housing.

In this context, the relationships between the construction tradition and the introduction of new technologies and materials, carried out by these social displacements, generate an impact on the preservation of the heritage and cultural identity of the communities. When people return, they bring new experiences and ways of thinking from where they currently live, generating a rupture in their traditions. ^15^

Therefore, the absence of a study that evidences the value of the vernacular architecture of Rumicruz has led to the physical deterioration of the buildings and their loss in the collective memory of the inhabitants. ^14^ In addition, this lack of knowledge about vernacular techniques, added to the economic factor, is responsible for local people building their homes with materials foreign to the region, which has caused consequences such as the transport of materials from places outside the community, the premature deterioration of the constructions, the use of materials that lack characteristics that can provide comfort inside the house, generating a lack of air conditioning of the spaces and an excessive use of energy. It is necessary to emphasize that Rumicruz has only two seasons, wet and dry. According to meteorological records, Rumicruz is located in climatic zone number five, which corresponds to the cold climate zone. This is due to its altitude of approximately 3215 meters above sea level, located around several snow-capped mountains, one of which is the snow-capped Chimborazo, on whose slopes the community is located, which is why it receives a considerable amount of wind, generating cold thermal sensation. In addition, the precipitation is 214 mm. ^16^ The increase in temperatures, climatic variability and extreme weather phenomena require new buildings to be designed to adapt to extreme climatic conditions. Therefore, applying bioclimatic and sustainable principles helps dwellings to maintain a comfortable indoor environment.

In summary, in a world facing global challenges such as climate change and biodiversity loss, rural housing is an example of how the local and the ancestral can provide solutions to global problems. Only by integrating these practices within a sustainable development approach, it will be possible to build a future in which humanity lives in balance with its environment.

Therefore, this research aims to design a sustainable rural housing proposal for a cold climate zone, taking as a case study the community of Rumicruz, based on the environment design guidelines, architectural typology, materiality and construction, bioclimatic and efficient housing.

## 2. Methods

### 2.1 Research design

The research has a qualitative-quantitative approach. The qualitative component focuses on identifying the qualities of the sector through a bibliographic exploration of guidelines related to the relationship between architecture and environment, architectural typology, materiality and construction techniques, bioclimatic design principles and efficient housing criteria. This information provides a solid conceptual basis for the formulation of a sustainable proposal.

An analysis of architectural references in Ecuadorian páramo areas, such as the community of Rumicruz, was also carried out, highlighting strategies applied for the air conditioning of spaces, the materials used, their properties, their environmental impact and the cultural identity associated with these elements. In addition, a comparison of two houses in Rumicruz, built in 1989 and 2016 respectively, a vernacular house and a current house, was carried out, which allowed identifying key aspects related to their architectural identity, functionality, materials, construction systems and guidelines. These aspects were rescued and incorporated into the new proposal. ^17^ This analysis was carried out using Ecotec software, obtaining relevant information on the influence of the use of local materials with better inertia and thermal conductivity, which favors a more comfortable interior temperature.

The importance of proper orientation with respect to solar movement and prevailing winds, which contribute to a better use of thermal gains towards the interior of the house, was also highlighted. For these reasons, the 1989 vernacular house achieved a higher thermal comfort index compared to the house built in 2016.

On the other hand, the bibliography of Condor ^14^ was reviewed, who analyzes the spaces and their relationship with the environment in the vernacular housing of Rumicruz. This review facilitated the understanding of key aspects of the context and guided the observation during the fieldwork.

From the quantitative approach, the degree of affectation of the built environment was evaluated through a diagnosis of the study site. For this purpose, the main source used was the Development and Land Use Plan of the Calpi parish, which provides relevant numerical data on population, economic activities and risk situations. These inputs made it possible to establish an objective starting point for proposing efficient solutions that respond to the real needs of the community.

Based on the information obtained from both approaches, it was possible to carry out an adequate field investigation, which made it possible to contrast the bibliographic findings with the current reality of the place.

### 2.2 Type of research

The objective of this research is to develop an architectural proposal for sustainable rural housing in the community of Rumicruz, province of Chimborazo, that promotes the preservation of the cultural and architectural identity of the place. In addition, it seeks to solve current and future problems related to sustainability criteria in construction. To achieve this, a mixed approach is proposed, since it will allow obtaining qualitative data, fundamental to analyze the sector and understand its critical vision regarding architectural design. The quantitative approach will be used for the collection of numerical data, which will provide accurate information on the spatial needs, as well as the functional and bioclimatic quality of the dwellings. In this way, it will be possible to develop a proposal that integrates design strategies in relation to the environment, architectural typology, materiality, construction, bioclimatic and efficiency in housing.

### 2.3. Research level

The present research is situated at an exploratory and propositional level. The exploratory approach allows us to understand the characteristics of the rural environment of the community of Rumicruz, province of Chimborazo, through the qualitative analysis of vernacular architecture and the physical, cultural and climatic context. Through the bibliographic study, field observation and comparative analysis of two representative houses, one vernacular from 1989 and the other contemporary from 2016, the architectural elements, materials, construction techniques and thermal comfort criteria that have influenced the configuration of housing in the sector were identified. This exploratory phase was essential to diagnose the problems affecting the community, both in terms of sustainability and loss of architectural identity.

The propositional approach, on the other hand, is based on the findings obtained during the exploratory phase and aims to develop an architectural proposal for sustainable rural housing that responds to the real needs of the population. This proposal considers criteria of energy efficiency, use of local resources, adaptation to the natural environment and rescue of cultural values. It is based on bioclimatic design principles and on the analysis of quantitative data provided by sources such as the Development and Land Use Plan (PDOT) of the parish of Calpi, as well as on the results obtained through the use of Ecotec software, which made it possible to evaluate the thermal comfort of the houses analyzed.

Overall, the research level allows not only to identify and understand the current problems of the rural habitat in Rumicruz, but also to formulate viable solutions that integrate sustainability, appropriate technology and cultural relevance.

### 2.4 Research modality

The research is developed through the application of two complementary methods: inductive and analytical, integrated in the methodological-logical process for specific purposes. The inductive method was applied through direct observation of the study site during the field trips carried out in the community of Rumicruz. This observation made it possible to identify recurrent patterns and characteristics in the built environment, such as the use of local materials, the orientation strategies of the houses and the climatic conditions of the páramo. Based on these elements, preliminary guidelines were established that guided the development of the sustainable architectural proposal, adjusted to the real needs of the place. On the other hand, the analytical method was implemented through the review of specialized literature, the comparative analysis between a vernacular house built in 1989 and a contemporary house of 2016, and the use of Ecotec software to evaluate the thermal behavior of both typologies. This analysis made it possible to break down each architectural element and contextualize its influence on thermal comfort, spatial functionality and energy efficiency. The results obtained provided a solid technical basis to justify the design decisions made in the final proposal.

### 2.5 Research procedures and techniques

A diagnosis of the current situation of the area will be made through field studies. The bibliographic research technique will be used to collect data, which will provide information on the cosmovision, local architecture, vegetation, customs and available materials. The problems affecting the area will also be identified, as expressed by its inhabitants. With the information gathered, an analysis will be carried out that will serve as the basis for designing a plan adjusted to the situation and needs of the sector.

### 2.6 Study population

It has been decided to carry out the research in the head of the community of Rumicruz. To this end, we will initially gather information from the Development and Land Use Plan (PDOT) of the Calpi parish, complemented by a critical observation during the site visit. Subsequently, a diagnosis will be made to obtain specific data on the needs of the sector. In this process, a direct consultation was carried out with the Rumicruz community authorities, who have updated data from the last population census, which registered a total of 350 families. However, due to the temporary or permanent displacement of some members for study or work reasons, it was considered appropriate to hold discussions with a representative of each family nucleus present in the community. As a result, a representative sample of 80 surveyed inhabitants was obtained.

## 3. Results

### 3.1 Diagnosis

Following the suggested methodology, the analysis of the Rumicruz sector and based on the bibliographic information available in the Calpi Parish Land Use Plan and exploratory field research identified problems such as the accumulation of garbage, abandoned or half-built houses due to economic difficulties, soil degradation in land and streams, damage to crops and the introduction of materials that do not improve the comfort of the inhabitants. The community is linked to economic livelihood activities, which makes the house mainly a shelter for the night, while the corridor or doorway of the house is used as a social space for rest and conversation.

Likewise, the study was carried out on a total of 79 dwellings, that is, all the dwellings present in the community. The research was developed through the elaboration of urban-architectural analysis cards, dividing the community into five sections. In each section, a compendium of the characteristics observed in the corresponding houses was compiled, including aspects such as materiality, typology, vegetation, symmetry, continuity, among others.

From an urban-architectural point of view, 95% of the dwellings are single-story, mostly built with block, tile, glass, brick and reinforced concrete. The roofs are flat and accessible, while the windows are small, limiting the entry of natural light and reflecting a lack of consistency in design between dwellings.

The urban analysis also revealed that the one-story rural dwellings are isolated, with horizontal growth, lacking symmetry and continuity, and surrounded mainly by vegetation consisting of shrubs and eucalyptus trees.

The vegetation at the study site is divided into four zones: páramo, natural forests, production zones, and abandoned areas and ravines, with the production zone being the most extensive. Native vegetation includes species such as eucalyptus, which is used in the construction of vernacular housing and in community water piping networks. The population of Rumicruz is located in the production zone because it covers the entire consolidated and built-up area.

On the other hand, according to the application of the field research, there is a marked contrast between the materials used in the current dwellings and in the vernacular dwellings of the area. The current dwellings have a predominance of concrete in the foundations, walls, structure and roof. They usually have two or more stories, are arranged contiguously and their roofs are accessible. These constructions use materials acquired in the city and a mixed labor force, as personnel are often hired from the city.

On the other hand, vernacular housing was mostly built with earth, wood, straw and reeds. It was built in isolation on the land, with a one-story typology, sloping roofs, and was built with local materials and labor.

On the other hand, some sustainable and/or ecological bioclimatic strategies that can be highlighted in the rural dwellings of Rumicruz focus on capturing solar gain to increase the interior temperature, due to the cold climate of the area. In vernacular dwellings, the use of openings was avoided to reduce heat loss as much as possible, and materials with high inertia and thermal conductivity were used, capable of capturing heat and transmitting it to the interior. An example of this is the use of earth, the predominant material in the walls of the vernacular dwellings studied.

In addition, the longest sides of the house were oriented towards the east and west, in response to the sun’s path in this geographical area.

Traditional construction techniques observed through field research, and which could be rescued for application in current community housing, include the use of local materials such as earth, straw, wood and stone. These materials offer several benefits, among them: the recovery of architectural identity, the use of resources with a smaller ecological footprint, better thermal properties and, consequently, greater thermal comfort inside the house.

Also, according to the observation of the architecture in the community of Rumicruz, the current houses present a modern architectural style, more linked to the urban than to the rural. This is evidenced by the use of concrete beam-column type structures, the presence of glass in the windows, accessible concrete slab roofs, as well as the use of steel in doors and in the protections of some windows.

### 3.2 Bioclimatic Requirements Analysis for exteriors and interiors

In the second stage of the research, an analysis of the bioclimatic requirements for the exterior and interior spaces of the study area is executed to make informed decisions during the design process. This will optimize thermal comfort and energy efficiency.

**Figure 1.**
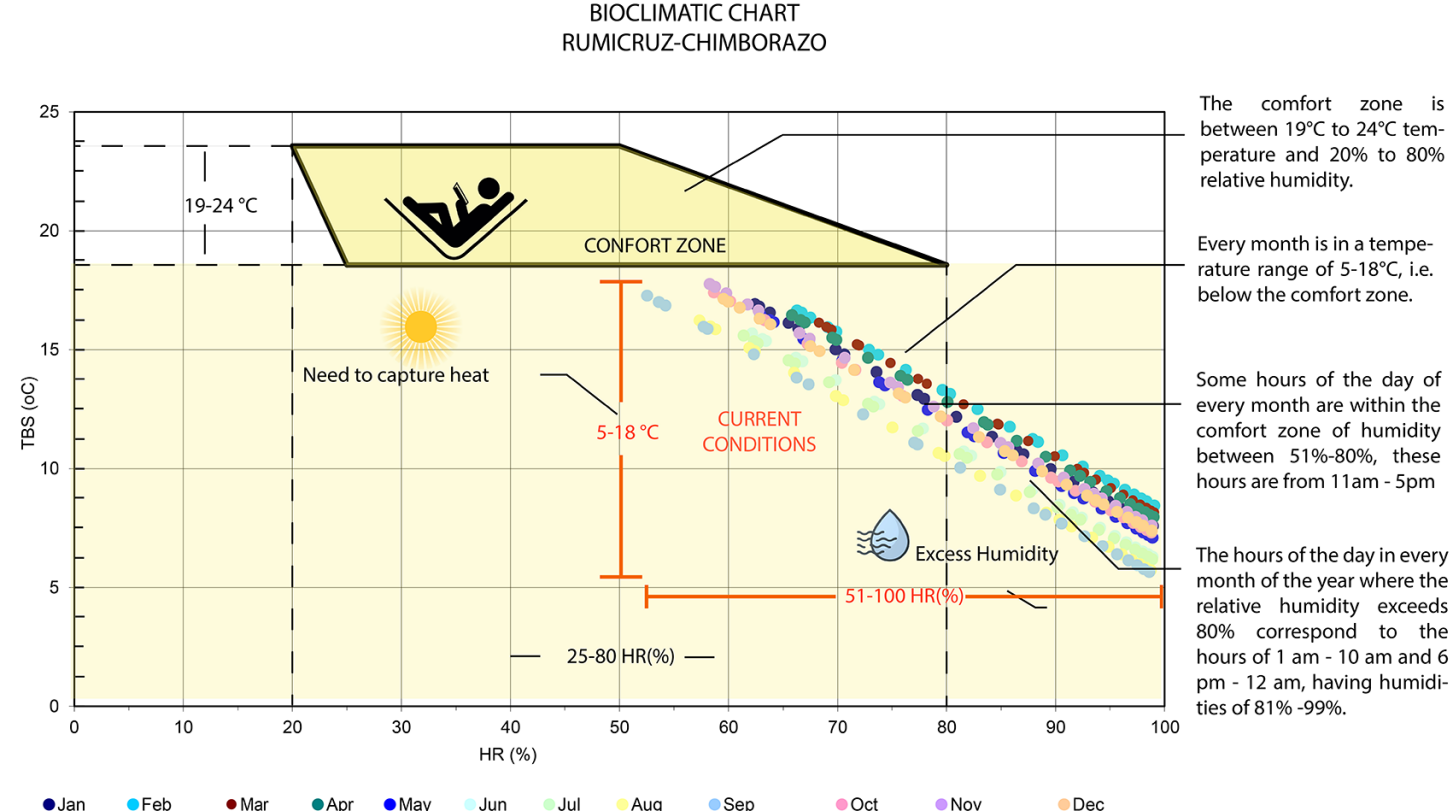
Rumicruz analysis with the Olgyay Chart ^18^ to determine thermal comfort requirements. ^38^

Figure 1 shows the Olya bioclimatic chart, applied to Rumicruz, includes a comfort zone with the ideal temperature and humidity values for outdoor environments, in which the ideal temperature values range between 19 and 24 degrees Celsius, and the relative humidity between 20% and 80%. ^18^ In the case of Rumicruz, all points are outside the comfort zone, presenting temperature ranges from 5 to 18 degrees Celsius and relative humidity from 51% to 100%. This indicates that during all months of the year, it is required to capture heat, emphasizing the need to use materials, colors, and textures with high thermal inertia to absorb heat and avoid losing it.

In the second phase of the research, an analysis of the bioclimatic requirements of the exterior and interior spaces of the study area is carried out in order to make informed decisions during the design process. In this way, thermal comfort and energy efficiency will be optimized.

**Figure 2.**
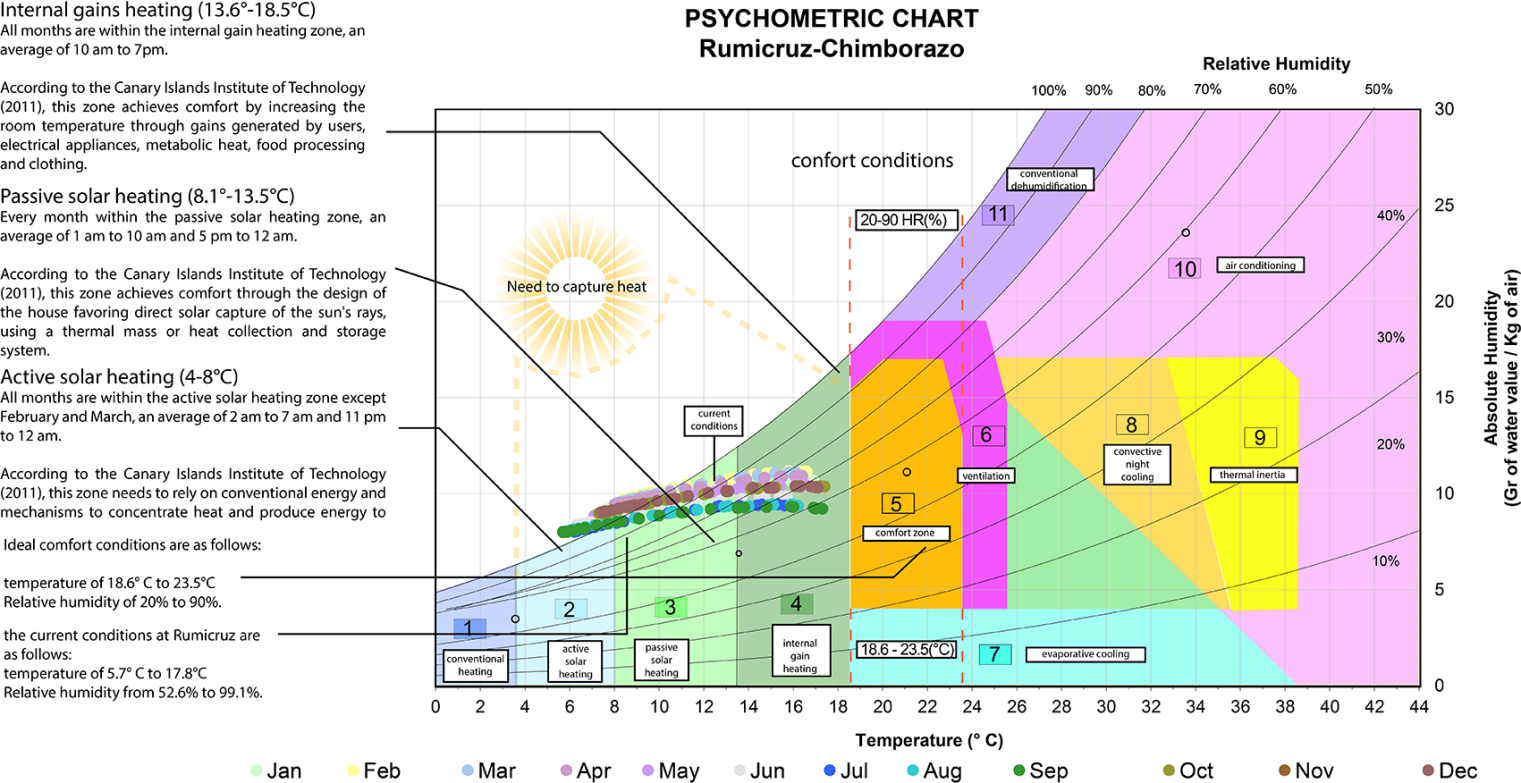
Analysis of the Givoni Bioclimatic Chart ^19^ applied to Rumicruz with Excel and Illustrator. ^38^

The psychometric chart of Givoni, ^19^ also known as the design chart for interiors, establishes the ideal temperature values between 18.6 and 23.5 degrees Celsius and relative humidity between 20% and 90%. ^19^ Throughout the year, Rumicruz does not present data within the comfort zone, since the temperature range goes from 5.7 to 17.8 degrees Celsius and relative humidity from 52.6 % to 99.1%. Therefore, corrective methods are needed to capture heat:

• Active solar heating: With temperatures between 3.8 and 8 degrees Celsius, a heating system is required using additional mechanical or electrical devices, such as heat exchangers, to capture, store, and distribute this energy efficiently, ensuring that the heat is adequately distributed even in adverse weather conditions or when solar radiation is insufficient. ^20^
• Passive solar heating: For temperatures between 8 and 13.5 degrees Celsius, systems that capture and distribute solar energy passively without the need for mechanical or electrical equipment to heat the interior spaces are applied. ^20^
• Internal gain heating: With temperatures between 13.5 and 18.5 degrees Celsius, comfort is achieved through heat generated by occupants’ activity and electrical equipment to maintain a comfortable temperature. ^20^ Figure 2 shows the following:

In this analysis, two graphs are presented: the bioclimatic chart and the psychrometric chart, which basically provide guidelines for the ideal comfort level in exteriors and the second allows us to have an approach to the type of strategies that should be applied in the house as such. Thus, the characteristics that the strategies proposed should have in order to have an ideal comfort level are defined in a general way and with the bibliographic review of Deffis, ^21^ some examples are established. However, this affects the Rumicruz housing in a positive way because it provides a series of responses to the adjacent climatic conditions, sustainable solutions that can be applied according to the needs and creativity of the users.

### 3.3 Proposal

The diagnosis made in the community of Rumicruz indicates that approximately 60% of the families are made up of four members: father, mother and two children. Therefore, the proposal considers a family of four as the main users. According to the Plan de Desarrollo y Ordenamiento Territorial (PDOT) of Calpi (2019), the main economic activity is agriculture. The father is also a bricklayer, a job that many Rumicruz residents do in nearby cities such as Riobamba. The mother takes care of household chores and is also involved in agriculture, while the children, aged 14 and 12, attend school and help with agricultural work, a common practice in the rural area. The needs identified for the family include areas for planting crops on the property and outdoor and recreational spaces, as well as specific spaces for the children to study and do their homework, as they currently have to use common areas such as the dining room or living room. The daily workday begins very early, between 4 and 5 a.m., so it is necessary for the home to be adequately air-conditioned from early in the morning. Throughout the day, the members of the household spend most of their time on work, academic and domestic activities, leaving little time for leisure. Agricultural activities involve daily trips of between 2 and 5 kilometers, often on foot, due to the lack of adequate areas for cultivation within their properties. This situation also affects the school performance of the children, who must help with the farm work and travel distances that take time away from their studies. Finally, the house is most occupied at night, from 5 p.m. to 5 a.m., so it is a priority to ensure efficient indoor climate control during these hours. To this end, we seek to maximize direct and indirect thermal gains during the day, taking advantage of solar radiation to heat the interior spaces.

**3.3.1 Relationship with the environment**

This indicator corresponds to the study of the property, the insertion of the house, its relationship with the landscape through the topography where it is located, the connectivity and accessibility networks between the property and nearby places, and the organization and collectivity at the social level.

**Figure 3.**
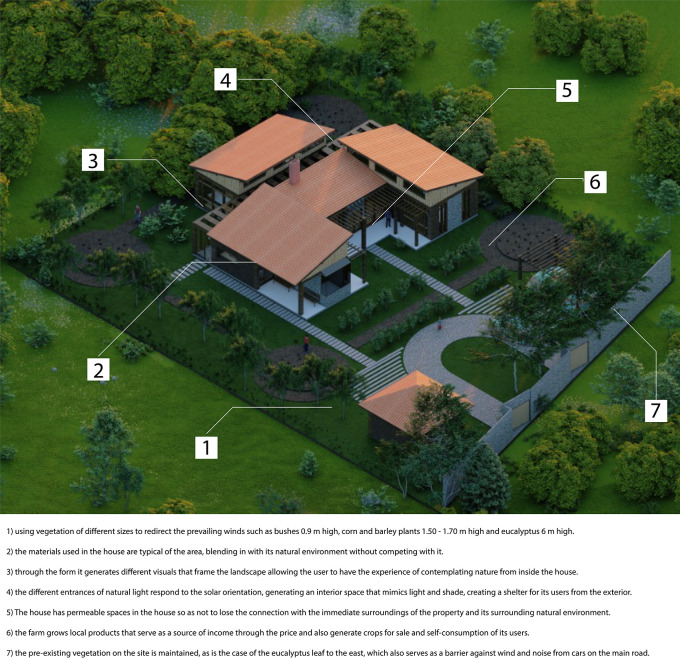
Environment guidelines for sustainable rural housing in Rumicruz-Ecuador, with ArchiCad, Lumion and Adobe. ^38^

• Sustainable rural housing proposal Rumicruz-Ecuador: The relationship with the environment is one of the main aspects addressed in the proposal. As shown in Figure 3, the house is inserted into the property in an isolated manner, avoiding damaging the views of both the project and the nearby houses.

Proximity to other dwellings is limited to two: one located to the north, 10 meters away, and the other to the west, 12 meters away. These distances are considered prudent to avoid disturbances related to noise generated by the daily activities of each dwelling.

On the east side of the house, there are eucalyptus trees native to the area, which have not been introduced by the proposal. For this reason, the project respects this natural condition, avoiding altering the environment.

To the south and west, the site had herbaceous vegetation of approximately 0,9 meters in height, which has been preserved for its natural value. In addition, this vegetation is managed as a barrier that protects the house from the prevailing winds, reducing their direct incidence.

In the northern zone, the vegetation has also been managed so as not to exceed 3 meters in height, thus allowing a direct view of the snow-capped Chimborazo.

As can be seen, the proposal incorporates vegetation proportional to the human scale. In the walkways of the project, vegetation has been chosen to avoid obstructing the views of users or interfering with the entry of direct natural light.

Another strategy implemented is related to the house’s crops, which are not concentrated in a single determined space, but form an integral part of the landscape. This design follows a cycle from planting to harvest storage.

**3.3.2 Typology**

This indicator focuses on the analysis of the housing program, considering for whom the project is intended, as well as its form and function. It also evaluates the progressive development of housing overtime and its integration with productivity-related aspects.

**Figure 4.**
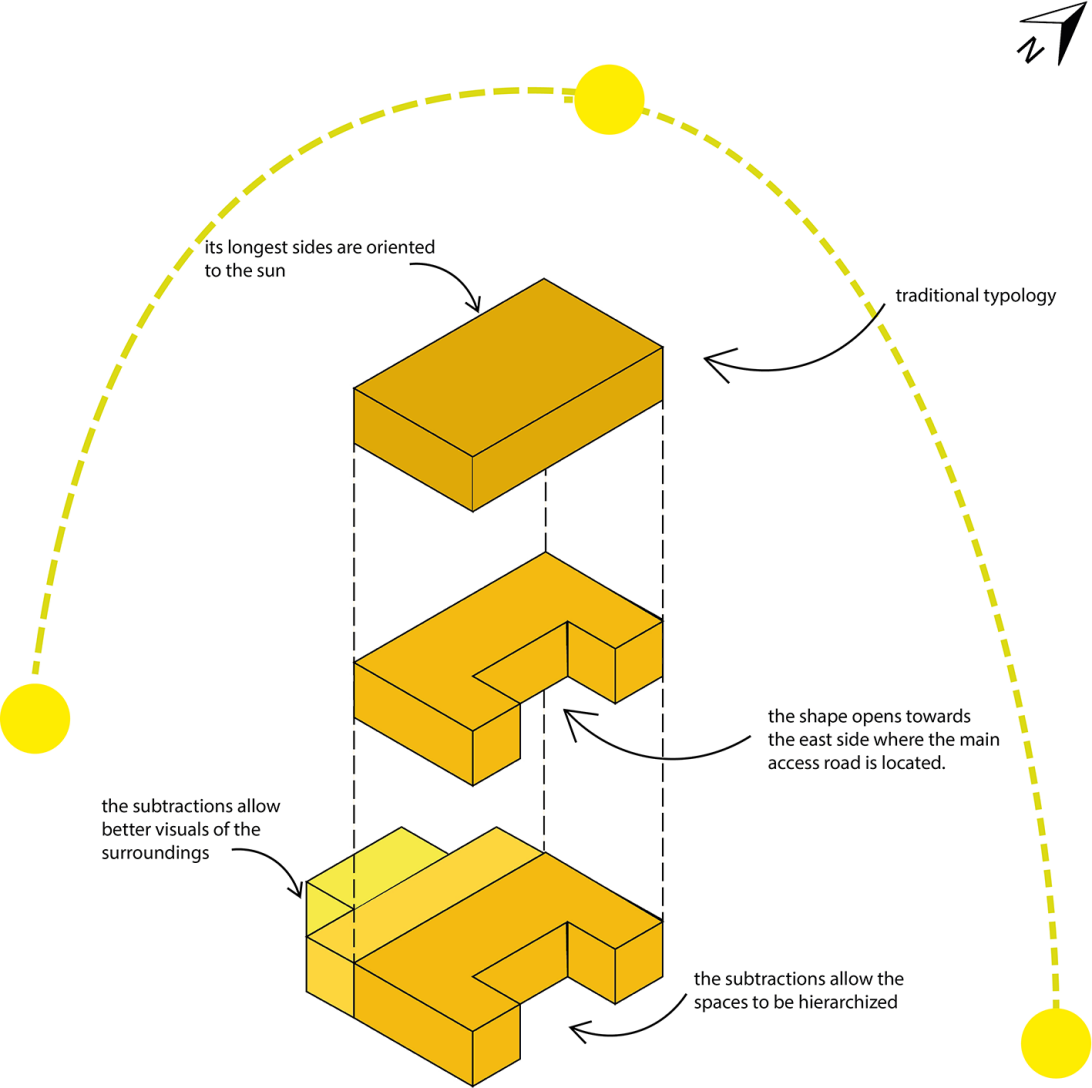
Typological guidelines for sustainable rural housing in Rumicruz-Ecuador, design with Adobe Illustrator. ^38^

• Sustainable rural housing proposal Rumicruz-Ecuador: The reference typology for this house is the traditional rural house of Rumicruz, which, in turn, responds to the typology of rural housing in Ecuador. This is characterized by the incorporation of an interior courtyard or form subtractions to create open spaces to the exterior. Figure 4 shows that the main shape of the house is a rectangular prism. This presents a subtraction to the east side in order to maximize the capture of natural light and heat during the morning. Additionally, two smaller rectangular prisms are added, strategically located to generate intermediate spaces that allow good lighting and heat gain on the west side during the afternoon.

As can be seen, the living spaces are organized according to their level of privacy. In the center is the social area, composed of the living room and dining room, which act as the center of the family union. To the south are the private spaces related to domestic activities, such as the kitchen, laundry, and clothes drying area. On the west side are the children’s bedrooms, while to the north is the master bedroom.

The house is designed so that users can fluidly move through the spaces. This design is complemented by a longitudinal corridor that facilitates direct and indirect circulation within the house.

Finally, the resulting form of the house not only optimizes its functionality, but also provides aesthetic and hierarchical value. The integration of volumes of different dimensions allows the spaces to be easily recognizable from the outside, achieving a clear differentiation and visual harmony, as shown in Figure 4.

**3.3.3 Materiality and Construction**

This indicator corresponds to the use of local materials, structure, envelope and construction processes.

**Figure 5.**
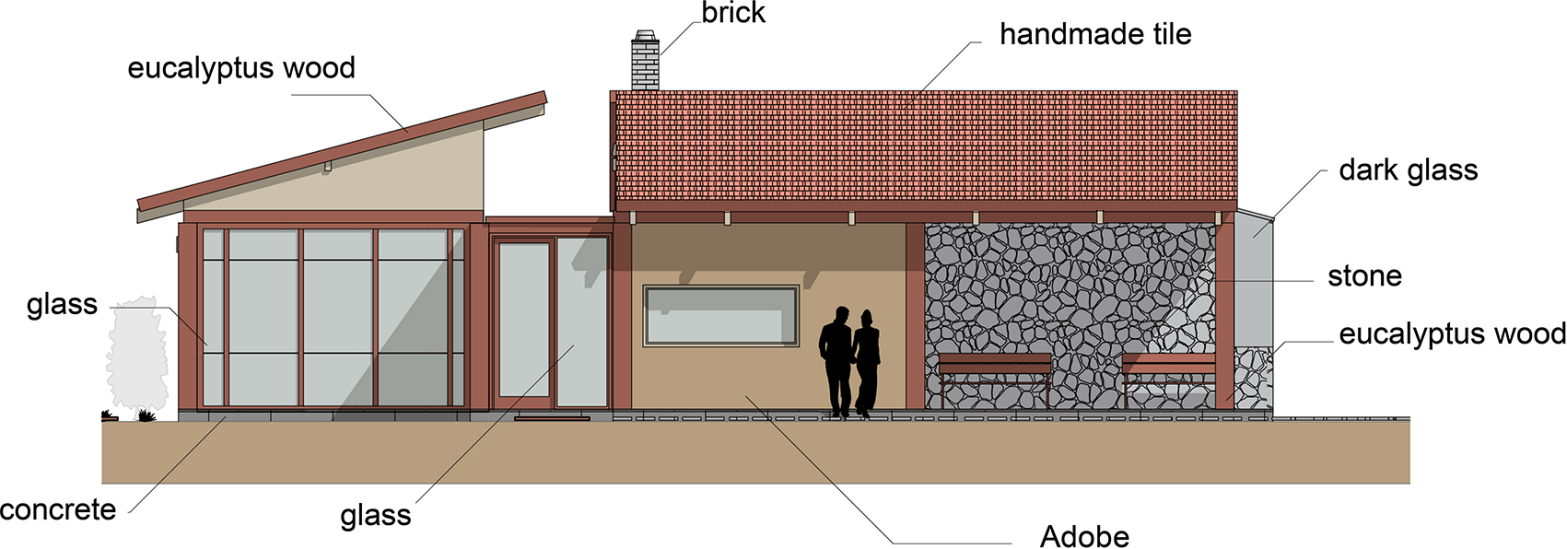
Materiality and construction guidelines for sustainable rural housing in Rumicruz-Ecuador, design with software. ^38^

Flexible modular housing proposal Cebadas-Ecuador: The housing materials have been selected according to the project conditions, prioritizing those of the area that adapt to the thermal and functional needs of the users. The materiality is detailed as follows:

• Foundations: Cyclopean concrete, composed of concrete and river stone, to ensure stability and durability.
• Flooring: Laminated wood that provides warmth and comfort to the house interior.
• Walls: Vary according to space. They include mudbrick walls, which offer excellent thermal insulation, concrete block walls filled with mortar covered with stone, to provide greater insulation and resistance.
• Windows: Wooden frames, according to the local style and materials.
• Fireplace: Built with a brick structure, providing functionality and aesthetics to the design.
• Roof: Made of eucalyptus wood and handmade tiles, materials native to the region that ensure thermal protection.
• Furniture: Also designed with eucalyptus wood, maintaining coherence with the constructive elements of the house. As shown in Figure 5.

Through the implementation of the architectural proposal with materials from the area, its construction is promoted through local labor, which promotes the economic development of the community members and, at the same time, allows for the conservation and transmission of knowledge about this type of construction to future generations, by integrating them into the construction process.

Within the proposed property, specific areas are defined for the cultivation and harvesting of local products such as potatoes, barley and corn. These products will later be stored in an infrastructure located on the same land, designed for storage, allowing them to be marketed both in the locality and its surroundings. This dynamic generates a network of economic sustenance for the users of the house and other members of the community. In addition, these products can be used for self-consumption, which contributes to guaranteeing the food sovereignty of its inhabitants.

**3.3.4 Bioclimatics**

It focuses on the climatic analysis of the place where the house will be built, psychrometric analysis, thermal comfort, lighting, and application of bioclimatic strategies.

**Figure 6.**
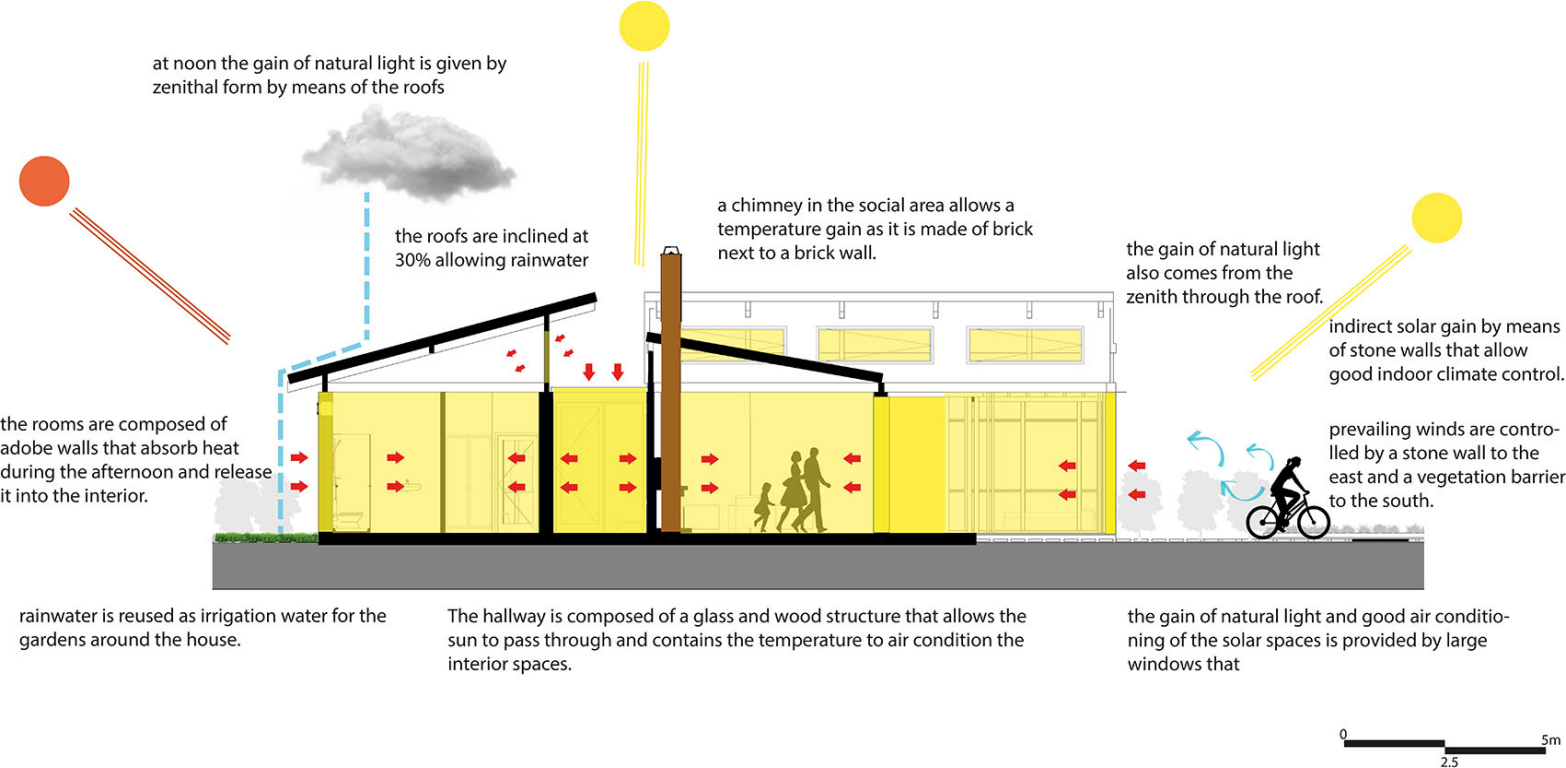
Directrices bioclimáticas para vivienda rural sostenible en Rumicruz-Ecuador, diseño con ArchiCad, SketchUp e Illustrator. ^38^

• Flexible modular housing proposal Cebadas- Ecuador: One of the main objectives in this proposal is the application of bioclimatic strategies in housing to reduce the consumption of resources and make the most of the natural conditions that the site provides. As shown in Figure 6, a series of strategies were applied that work in conjunction with the activity to be developed in each space and with the shape of each one of them.
  Among the main strategies are the direct and indirect gain of solar radiation to warm the spaces, the incidence of natural light through permeable and semi-permeable elements, and the entry of natural light from the zenith. In addition, three spaces have been proposed that, in addition to contributing to the air conditioning of the spaces, also constitute elements of energy efficiency by contributing to the reduction of electrical energy consumption, these strategies can be seen below.

For the implementation of this strategy, the corridor has been considered as a connection point between the different spaces of the proposal, as well as a transition area between the social section and the bedrooms. In addition, due to its location, the corridor receives solar radiation at midday, when the temperature is at its highest.

Taking into account these conditions, a heat trap strategy has been designed, consisting of a semi-permeable space of glass and wood, which allows the passage of natural light into the interior of the house and, at the same time, contributes to the increase of the interior temperature by capturing heat in mudbrick walls, which have high thermal inertia. These walls absorb heat and gradually release it into adjacent spaces.

To optimize thermal capture and insulation, a chamber has been added to the floor of the structure, composed of concrete, river stone, and a non-slip laminate glass covering. This chamber functions as a permeable space that absorbs temperature through the stone, retains it, and distributes the heat through PVC pipes connected to adjacent spaces. Pumps extract the heat from the chamber, channeling it to the living room, dining room, and bedrooms respectively.

**Figure 7.**
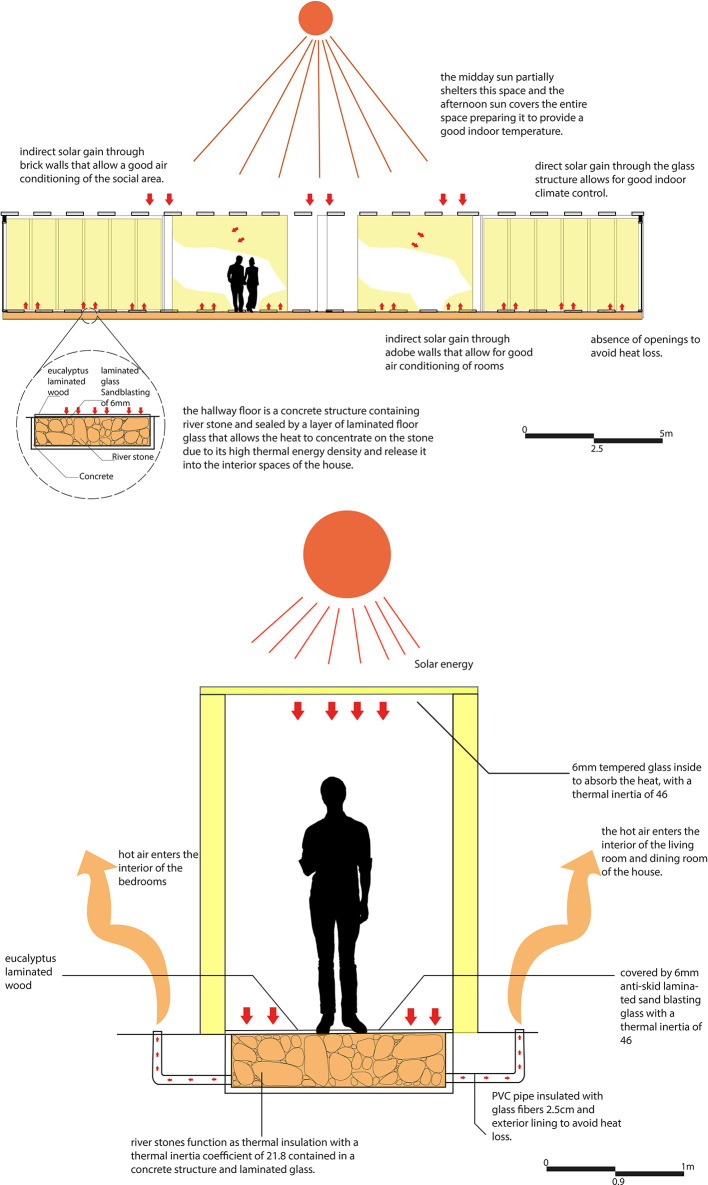
Heat gain in rural houses in Rumicruz with ArchiCad software, and Adobe Photoshop. ^38^

The floor of this corridor is composed of a combination of materials that alternate between non-slip glass and eucalyptus wood slats. This choice is intended to provide greater safety for the user when walking through this space. However, the bioclimatic strategy is not affected, maintaining its efficiency and functionality by continuing to store heat and dispersing it to the adjacent spaces. As shown in Figure 7.

For this strategy, the main objectives are to air-condition the spaces and reduce electrical energy consumption in the proposal. In this sense, a solar clothes dryer has been designed, consisting of a chamber formed by a gravel floor, stone walls, and glass, which allows the entry of natural light and solar radiation.

The operation of the system is based on capturing solar radiation through the glass, heating the space with the help of the black-painted gravel floor. In addition, pipes filled with sand and painted black on the outside are incorporated to maximize the temperature inside the space, favoring the drying of clothes. To reduce humidity, cool air is allowed in from the winds, which pushes the warm air into the adjacent spaces (laundry and kitchen), helping to keep them warm while the clothes dry.

**Figure 8.**
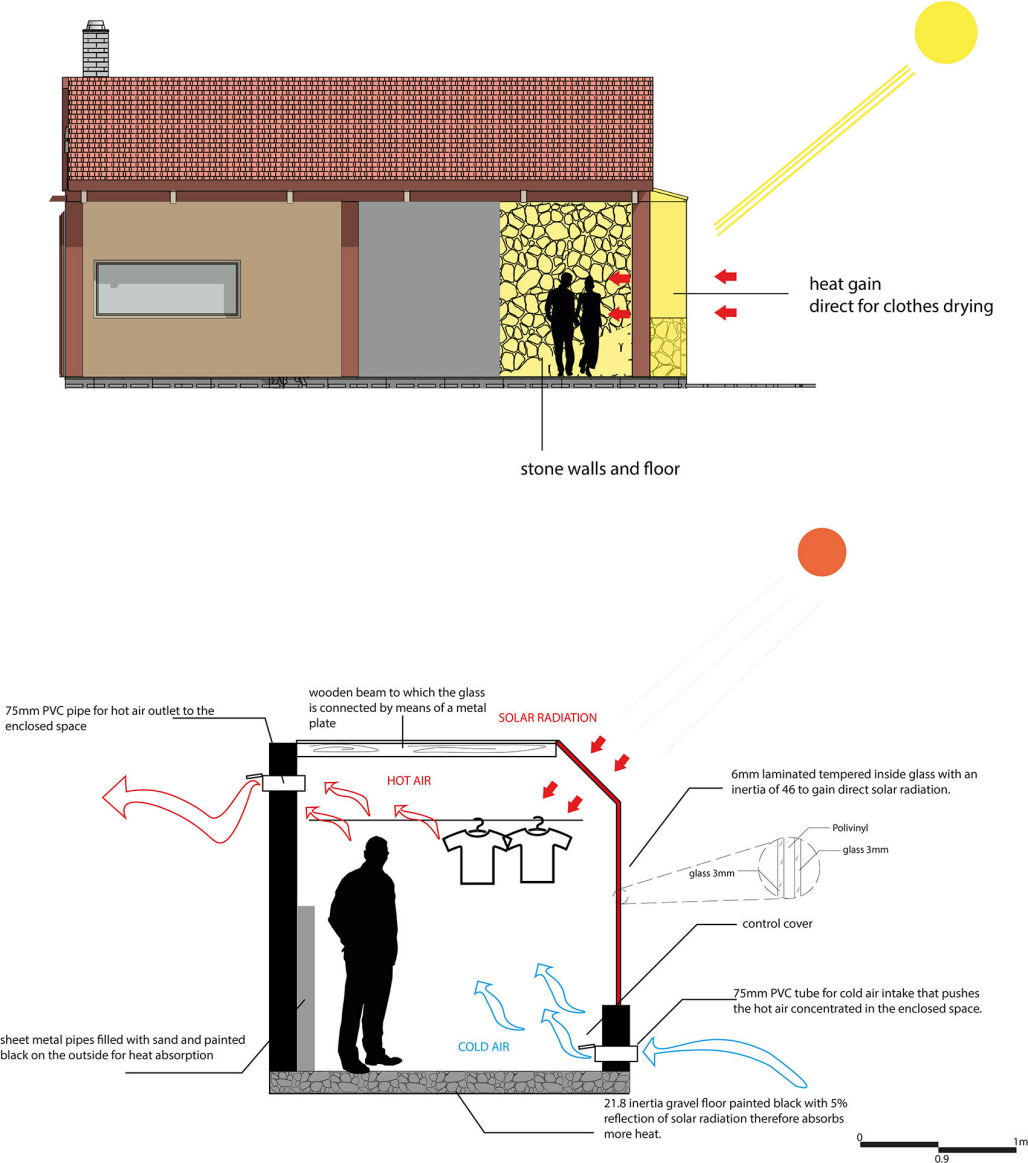
Natural dryer proposed for sustainable rural housing in Rumicruz with ArchiCad and Photoshop software. ^38^

In this way, this strategy not only helps to air-condition the space and adjacent spaces but also reduces the need to use an electric dryer, which is especially useful given that frequent rainfall in the area makes it difficult to dry clothes. As shown in Figure 8.

**Figure 9.**
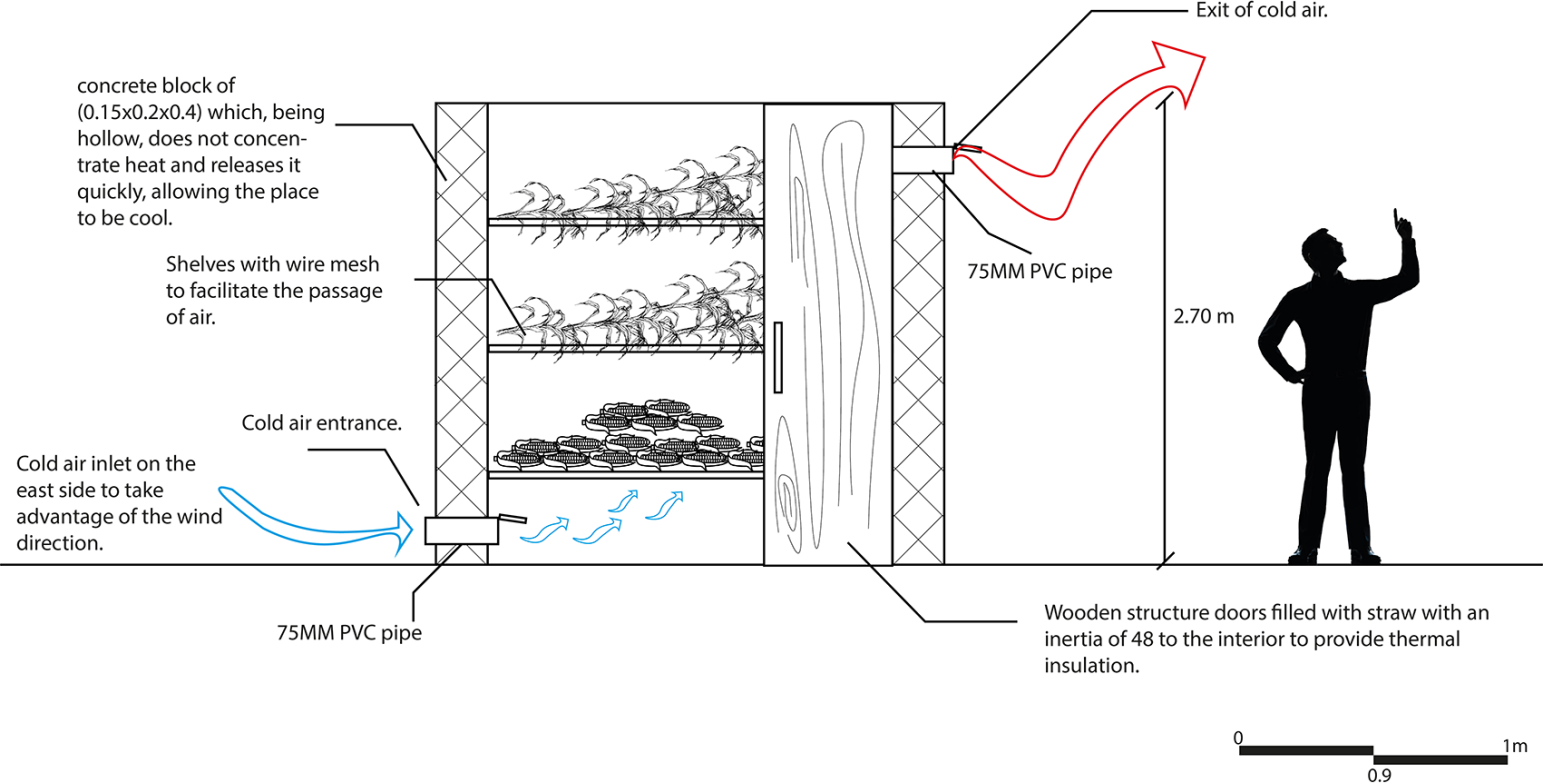
Proposed crop storage for sustainable rural housing in Rumicruz with Archicad software. ^38^

As can be seen in Figure 9, this strategy is aimed at reducing electricity consumption and prolonging the use of the productive resources of the proposal, which has a positive impact on its economy. A natural cooler has been designed in the space destined for the collection of harvest from the crops outside the property, in order to support one of the main economic activities of the house user.

The operation of this strategy consists of creating a shelf for storing the harvest, which is attached to the wall on the east side of the space since the prevailing winds come from that direction. This allows cold air to enter from below through a PVC pipe, while warm air is expelled to the outside, keeping the products cool. To help this air flow, the shelves are composed of wire mesh that facilitates the passage of cold air through them.

This strategy contributes to the reduction of electric energy consumption and reduces the need to purchase a refrigerator to keep products fresh, which has a positive impact on both energy efficiency and the economy of the user as well as the proposal.

## 4. Conclusions/Discussion

The results of this research are based on providing the user with the ideal thermal comfort inside the house, so that the user feels comfortable in the performance of their activities. ^22^ For this purpose, several case studies of existing houses in the area were taken as a reference in order to evaluate their characteristics and their response to thermal conditions.

The analysis was carried out by comparing two houses of the Rumicruz community, built in 1989 and 2016, respectively: a vernacular house and a current house. From this comparison, key aspects related to their architectural identity, functionality, materials, construction systems and guidelines were identified, which were rescued and incorporated into the new proposal. ^17^

The study used Ecotec software, through which relevant information was obtained, highlighting the influence of the use of local materials with better inertia and thermal conductivity, which allow maintaining a more comfortable indoor temperature. Likewise, the importance of an adequate orientation of the house with respect to solar movement and prevailing winds was highlighted, which favors a better use of thermal gains towards the interior of the building.

For these reasons, the 1989 vernacular house achieved a higher thermal comfort index compared to the house built in 2016.

With the purpose of performing a climate analysis, it was necessary to consider the use of various tools, such as gadgets, measuring devices, indexes, diagrams and bioclimatic simulation software. These tools allow analyzing an element in 3D and calculating its performance in relation to physical phenomena, material properties, space occupation, and applied strategies. ^23^ Thus, by using the Ecotec software, a program that allows the designer to have an approach to the energy performance of the building through the analysis of the project, ^24^ each of the selected houses was analyzed taking into account their location in the community, the orientation and the materials.

**Figure 10.**
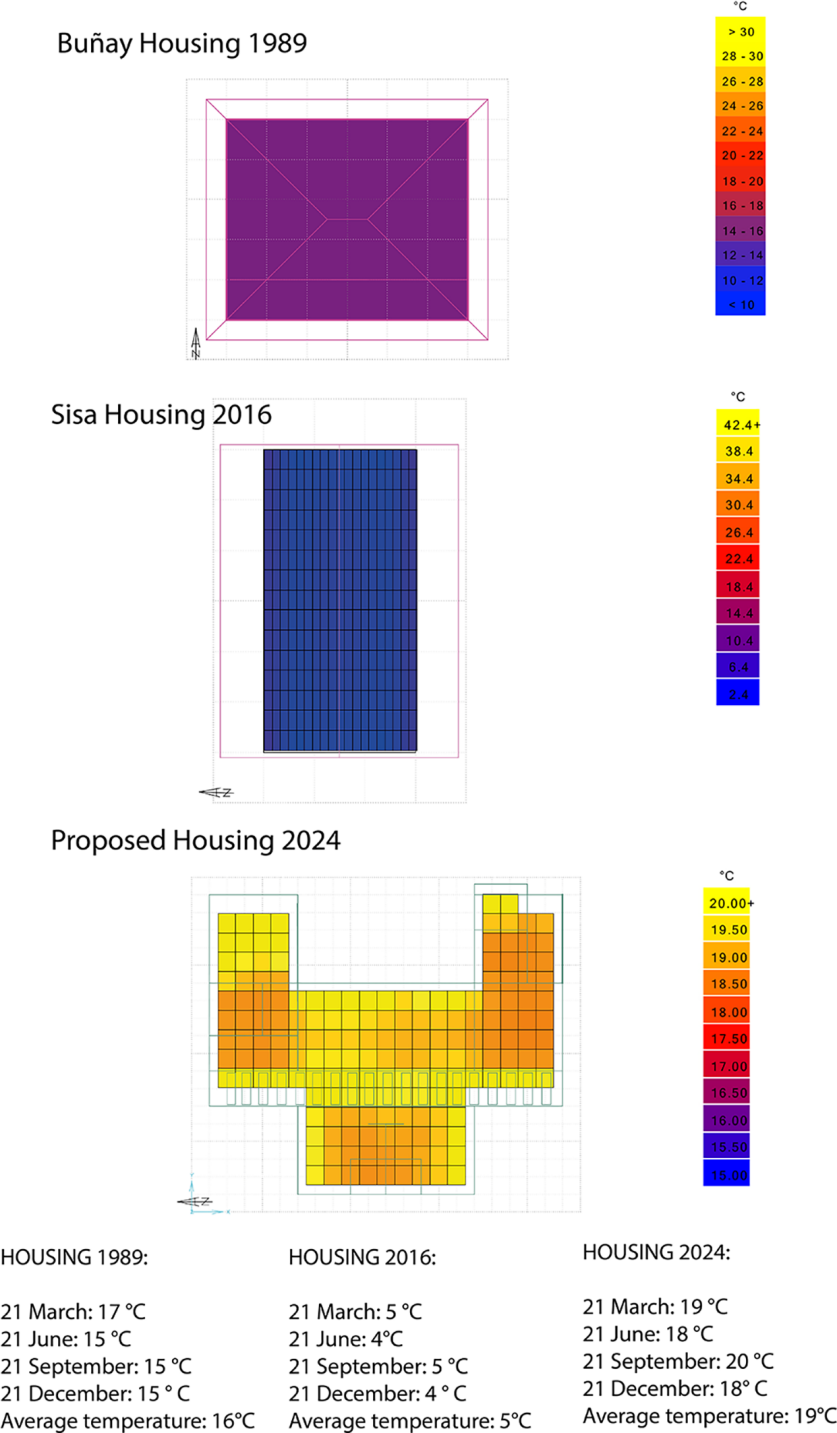
Comparative of Architectural plan analyzed using Dynamic Daylighting. ^38^

The average temperature results inside the houses were compared on the most critical dates of the year. Therefore, Figure 10 shows that:

• For the 1989 house, the average indoor temperature is 16°C, i.e., 2°C below the ideal comfort range.
• For the 2016 house, the average temperature experienced indoors is 5°C, i.e., 13°C below the ideal comfort range.
• For the house proposed in this research, the average indoor temperature is 19°C, which is 1°C above the ideal comfort range. As shown in Figure 10.

These results show that the proposed house increases the temperature inside, thus achieving the ideal parameters of thermal comfort in Rumicruz. This is the result of using passive strategies such as the appropriate orientation since direct and indirect solar gain ensures the concentration of a good indoor temperature in the house. ^25^ These solar radiation inputs can be opened or closed according to the user’s needs, i.e., if it is necessary to concentrate the temperature, the windows or openings will be kept closed, and if it is necessary to cool the space they can be opened or partially opened. ^26^

It is necessary to consider that an adequate location taking into account the prevailing winds permits a natural ventilation system and cools the house, also a proper connection with the environment allows it to be directly linked with the sociocultural context of the place. ^27^ Therefore, the proposal considers the activities of the users, their way of thinking, and the relationship with the space. Regarding the typology of the building, the traditional Rumicruz housing is taken into account, built in a dispersed manner on large plots of land in order to have space for raising animals and agricultural activities. ^28^ Since the house is in a rural context, it must also include space for productive activities carried out by some or all members of the household. ^29^ Therefore, it is essential to start from a previous diagnosis of the site and the user. In the next phase, it is necessary to create a program based on the results, their requirements, activities as well as climatic conditions of the site ^30^ which determined the need to include spaces for planting and harvesting the vegetation already present in the property and its mountainous environment.

It is also important to work with suitable materials for the climatic variables of the place. ^31^ Since climatic factors and natural elements have an impact on the materials and can improve or reduce their behavior, i.e., they can increase the thermal comfort in a space or decrease it. Thus, the proposal is based on a mixture of materials at the ground level (concrete, river stone, wood, glass), at the wall level (mudbrick, concrete blocks filled with straw, brick), and at the roof level (wood and tile).

Sustainable architecture should reduce the consumption of resources, so local materials were employed, which were also used in the vernacular houses of the area, such as: eucalyptus wood, straw and river stone. ^32^ These vernacular houses make use of materials according to the climatic conditions or floors on which they are located, responding to the environmental needs of the area. ^33^

Therefore, the climatic conditions of the Rumicruz community have been evaluated, obtaining as a result the needs and requirements for the proposal, so it is considered of vital importance the application of bioclimatic tools to obtain preliminary data of the place, being the most used: the bioclimatic chart of Olgyay and the psychrometric chart of Givoni. ^34^

Using these resources in Rumicruz permits determining the requirements to improve the thermal comfort of the house. However, it is important to consider that the results will be defined based on the designer’s criteria and that the best option is to perform a combination of strategies as suggested in the proposal. ^35^ Besides having an approach to the requirements and the application of these, the following strategies are applied: passive solar heating (heat traps, solar heaters, heat collector walls), active solar heating (heater), and solar heating by internal gains (heat produced by users, appliances, kitchen, stove) that allow the use of natural resources, materials of the area and electrical devices that contribute to the increase of temperature in the house. ^21^ However, the need to apply a sustainable design in housing also includes the application of energy efficiency strategies to reduce the high energy consumption of conventional energy sources such as electricity. ^36^ It is also considered essential that buildings have guidelines that promote the reduction of energy consumption through individual strategies that are adapted to the specific needs and conditions of each project. Focusing on the concentration and dispersion of heat to reduce the use of electric heating, taking advantage of air movement and passive cooling of spaces, air conditioning of internal spaces, water management and its reuse as well as the use of natural light. ^37^

The conclusions obtained in the research are as follows:

• Through the diagnosis of the community of Rumicruz, several aspects of the place have been identified, such as: architecture, culture, vegetation, available resources, climate, economy and activities of the people. In this way, a broader knowledge was obtained in order to propose the project in accordance with the needs and problems of the place.
• The proposal has taken into account everything previously studied and analyzed. The proposal has been made for a family of 4 members (father, mother, and two children) integrating the traditional and current activities in the community of Rumicruz, providing spaces for planting and harvesting products of the area, as well as spaces for daily life.
• Regarding the use of materials, a mixture of traditional mudbrick, stone walls, and concrete blocks filled with mortar has been used to preserve the identity of the place without neglecting the current needs of its inhabitants. This is how both traditional and modern construction systems can be applied, however, it should be considered that some have a better performance than others depending on the conditions of the project.
• A comparison was made in the Ecotec software between the traditional housing, the current housing, and the proposed housing where the expected results were obtained, having the proposal as the housing that meets a better level of comfort for the user, as well as a better thermal level throughout the year, increasing users’ quality of life without losing their identity. With this, it is possible to determine that there is no perfect housing model to be replicated to improve its inhabitants’ conditions, but an in-depth study is required to obtain the best response.

## Data Availability

No data are associated with this article. Zenodo. Sustainable Rural Housing in Cold Climates; A Model for Rumicruz-Ecuador. https://doi.org/10.5281/zenodo.14940270. ^38^ The project contains the following underlying data:
• Sustainable Rural Housing in Cold Climates; A Model for Rumicruz-Ecuador. (Image). Data are available under the terms of the Creative Commons Attribution 4.0 International license (CC-BY 4.0).

## Software availability

- Adobe Illustrator ( https://www.adobe.com/products/illustrator.html)
- Adobe Photoshop ( https://www.adobe.com/ec/products/photoshop/free-trial-download.html)
- ArchiCAD ( https://graphisoft.com/ec/solutions/archicad)
- Dynamic Daylighting ( https://andrewmarsh.com/software/daylight-box-web/)
- Lumion ( https://lumion.com/)
- Microsoft Excel ( https://www.microsoft.com/en-us/microsoft-365/excel)
- SketchUp ( https://www.sketchup.com/es)
