# Supplementary material for: Sustainable rural housing in cold climates: a model for Rumicruz-Ecuador
Source: F1000Res. 2025 Jul 29;14:427. Originally published 2025 Apr 11. [Version 2] doi: 10.12688/f1000research.162591.2 (PMC12648017; doi:10.12688/f1000research.162591.2)
Supplement: Peer review history

# Reviewer report: Reviewer response for version 2

**DOI:** 10.5256/f1000research.185309.r430821

**Published:** 2025-12-23

**Authors:** Elham K. Hassani

**Affiliations:** 1 Department of Architecture and Design, Faculty of Architecture, University of Sapienza, Rome, Italy

The authors present a mixed-methods, site-specific architectural design study for Rumicruz (Calpi parish, Chimborazo, Ecuador), where vernacular housing has been partly replaced by modern/urban construction forms. They combine field diagnosis (urban-architectural analysis of the community, interviews/surveys), literature review, and bioclimatic simulation (Ecotec / Dynamic Daylighting) to develop a sustainable rural house prototype for a four-person household. The proposal mixes vernacular materials (mudbrick, stone, eucalyptus wood, thatch/tile) and passive/active solar strategies (heat trap corridor, solar dryer, floor heat chamber with stone + pipe distribution). The manuscript reports comparative simulation results for a 1989 vernacular house, a 2016 contemporary house, and the proposed design (average indoor temperatures: 16°C, 5°C, and 19°C respectively), and argues the proposed design better meets thermal comfort in the local cold climate.

**2) What’s good / strengths**

- Clear research motivation: preserving vernacular identity while improving thermal comfort in high-altitude cold rural contexts is topical and relevant.
- Mixed methods approach (field diagnosis + simulation + typological analysis) is appropriate for an architectural-design case study.
- Practical design strategies are concrete, culturally sensitive and actionable (materials, heat trap, solar dryer, storage cooling). These are useful for teaching and design inspiration.

**3) Major issues that must be addressed (required before acceptance)**

**A. Data availability — provide the underlying data and simulation files (MANDATORY)**

 Problem: the paper’s Data Availability statement says “No data are associated with this article,” but the manuscript reports numerical simulation outputs and a community diagnosis. The Zenodo DOI given appears to contain only an image; there are **no raw climate files, Ecotect/Ecotec model files, Dynamic Daylighting files, input spreadsheets, or survey data** available in the public repository. Without those, the simulations and community claims are not reproducible.

 What authors must provide:

1. Upload to a trusted repository (Zenodo / institutional repository) and link from the article:
  - All Ecotec (or Ecotect — see B.3 below) model files (project file, geometry, materials, schedule, boundary conditions) used for the 1989, 2016 and proposed models.
  - Dynamic Daylighting / Daylight Box inputs and outputs used for Figure 10 (if used).
  - Raw climate/weather file(s) used for simulations (EPW or local meteorological station records) with source metadata (period covered, station, any processing).
  - Survey instrument and anonymized survey data (the 80 respondents / 79 dwellings data), urban-architectural analysis cards, and the spreadsheet(s) used for any quantitative summaries.
  - Any scripts, calculation spreadsheets (Excel) and figure source files needed to reproduce plots and the Olgyay/Givoni charts.
2. Update the Data Availability statement to describe exactly which files are available, what license, and how to access them.

Why mandatory: reproducibility and validation of the major claim (proposed house reaches ~19°C) require these inputs.

**B. Methodological clarity and transparency (MANDATORY / must be fixed)**

 Problems and required fixes:

1. **Study population & sampling**
  - The Methods / Study population text states information was taken from PDOT and that “a representative sample of 80 surveyed inhabitants was obtained” and “the study was carried out on a total of 79 dwellings” — but the design and sampling procedure is underdescribed: how were respondents selected (random, convenience, representative of household types), what questionnaire/metrics were used, when fieldwork happened (dates), who collected data, and how were urban-architectural analysis cards produced? Provide a concise Methods subsection with: sampling frame, inclusion/exclusion rules, survey instrument (or link to it in the repository), dates of fieldwork, and number of houses/dwellings studied.
2. **Diagnostics and measurements**
  - The manuscript describes findings (garbage, abandoned houses, vegetation zones). How were these recorded? Were GPS coordinates taken? Please add a map showing the 79 dwellings, the 6 preserved vernacular dwellings (with their dates: 1949–1960 as already stated), and the vegetation zones. Add a short paragraph describing observational protocols and any instruments used. Reviewers explicitly requested a map and clearer urban study methods — add them.
3. **Simulation details (critical)**
  - For each Ecotec / Dynamic Daylighting simulation provide: software version, geometry/occupancy schedules, internal gains, infiltration/ventilation rates, material thermal properties (density, conductivity, capacity), boundary conditions, meteorological file name and period, simulation timestep and period simulated (which “most critical dates” were used?), and any assumptions (e.g., closed windows at night). Also confirm whether “Ecotec” refers to **Ecotect** (commonly used) — several reviewers pointed out spelling/terminology and this must be corrected and clarified. Without these inputs the reported average temperatures (16°C, 5°C, 19°C) cannot be independently evaluated.
4. **Validation / sensitivity**
  - Provide a short validation or sensitivity check: e.g., compare modelled indoor temperatures for the existing 1989 house to on-site measurements (if available) or run sensitivity analyses to show how robust the 19°C result is to reasonable variations in material properties, infiltration rate, or meteorological year. If measurements were not taken, explicitly state that and discuss implications.

**4) Minor but important changes (strongly recommended)**

1. **Language, structure, and figure clarity**
  - Several reviewers flagged grammar, repeated words, and figure labelling (e.g., “Psychometric” vs “Psychrometric”, Ecotec vs Ecotect). Do a careful language edit (native speaker or professional copyedit). Correct figure captions and ensure high-resolution figures and legible axis labels.
2. **Terminology consistency**
  - Be consistent: “Ecotec” vs “Ecotect”; “psychrometric” spelling; “Olgyay” vs “Olgyay Chart” phrasing. Spell out acronyms on first use.
3. **Return conclusions to objectives**
  - Some reviewers asked that conclusions explicitly reflect which objectives were met and which were not, and list limitations (e.g., single-site study, lack of measured indoor temperatures). Add a short paragraph of limitations and future work.
4. **Provide construction/specification details for practitioners**
  - For the paper to be useful for builders/practitioners include a short appendix or extended data with typical wall/roof/floor build-ups (thicknesses, material properties, recommended assembly details) and an approximate bill-of-materials or cost/constructability discussion. This increases the teaching/practical value.

**5) Specific editorial suggestions tied to manuscript text/figures**

 (quote locations are page numbers from the uploaded PDF)

- **Page 4**: tighten narrative; remove duplicated “THUS” and other repeated words; check paragraph breaks.
- **Figures 1 & 2**: clarify whether Figure 2 is “Psychrometric” and provide numerical axis labels and the meteorological data period used to plot points.
- **Methods Section (2.5–2.6)**: explicitly state how the “urban-architectural analysis cards” were designed and what fields they contained (e.g., wall material, roof, roof slope, number of stories, year built). Add a sample card in extended data.
- **Results (3.1)**: clarify the 79 dwellings vs “350 families / 80 surveyed inhabitants” — explicitly state what each number refers to (households present, households surveyed, individuals surveyed).

**6) Assessment mapped to your original checklist items (expanded)**

**Is the background described in sufficient detail? — Partly**

- Why partly: the manuscript provides a reasonably good narrative of cultural loss, timeframe (since ~1985), and the presence of only six surviving vernacular dwellings (oldest 1949, newest 1960). However it lacks **deeper socio-historical detail** (e.g., which foreign construction models/materials arrived and why; more on migration drivers) and maps/visual evidence to support claims about the spatial progression of change. Add a short historical paragraph and citations, and provide the requested maps.

**Is the work clearly presented and does it cite current literature? — Partly**

- Why partly: the literature cited is relevant and reasonably current, but presentation issues (grammar, figure labelling, methodological placement of software descriptions) reduce clarity. Correcting the presentation and moving all simulation software details into Methods will address this.

**Is the statistical analysis and interpretation appropriate? — Partly**

- Why partly: the paper uses building energy/daylighting simulation rather than statistical hypothesis testing. Interpretation of simulation outputs is plausible, but the manuscript lacks sufficient detail about simulation inputs, assumptions and any uncertainty quantification. Authors should add these details and a sensitivity/validation step.

**Are all source data available? — No**

- Why no: the statement “No data are associated” contradicts the claims and the Zenodo entry appears to only include an image. The authors must publish the full set of underlying files (see Section 3.A above).

**Are conclusions supported by results? — Partly**

- Why partly: the simulation numbers do support the main thermal comfort claim, but the lack of reproducible inputs, lack of measurement validation and incomplete methods weaken confidence. After the authors provide model files, climate files and (preferably) a simple validation/sensitivity analysis, conclusions will be on firmer ground.

**Is the case useful for teaching/practitioners? — Partly**

- Why partly: the design discussion, strategies and images are pedagogically valuable. But to be fully useful in teaching/practice, the paper must include build-up details, construction drawings or component specifications, maps, and the reproducible data noted above.

**7) Specific list of required changes (summary for editors / authors)**

1. **Add a reproducible data package** to Zenodo (or equivalent) containing: simulation files, meteorological file(s), raw survey data (anonymized), urban analysis cards, spreadsheets and scripts used to generate figures. Update Data Availability statement accordingly.
2. **Expand Methodology**: precise sampling description, dates, instruments, urban analysis protocol, and number clarifications for households/individuals/dwellings (79 houses vs 350 families vs 80 respondents).
3. **Provide full simulation details and inputs** for Ecotect/Ecotec/Dynamic Daylighting: software name & version, material thermal properties, infiltration rates, occupancy and internal gains, boundary conditions, timestep, and any control strategies. Include model geometry files. Run and present a sensitivity/validation check or explain why it is not possible.
4. **Add maps and visual data**: site map with dwelling locations, preserved vernacular dwellings, vegetation zones, and the location of the case study property.
5. **Correct terminology, spelling and grammar**, and ensure figure captions are informative and figures are high resolution. Consider a language edit.
6. **Add construction-level details** (typical wall/roof/floor sections, thicknesses, recommended assembly) in main text or as extended data so practitioners can use the design.
7. **Revise conclusions** to explicitly return to research objectives and state limitations and next steps.

**8) Final recommendation (for editor)**

**Major revision required**. The manuscript has merit and presents useful design strategies, and the simulation results, if reproducible, support the central claim. However the lack of underlying data, insufficient methodological detail (sampling, simulation inputs) and presentation issues prevent acceptance in its current form. If the authors supply the data and address the methodological and editorial points listed above, the manuscript would be suitable for publication. Several prior reviewers made similar points and the authors appear to have addressed some items already — the outstanding issues are reproducibility/data and methodological transparency.

Is the case presented with sufficient detail to be useful for teaching or other practitioners?

Partly

Is the work clearly and accurately presented and does it cite the current literature?

Partly

If applicable, is the statistical analysis and its interpretation appropriate?

Partly

Are all the source data underlying the results available to ensure full reproducibility?

No

Are the conclusions drawn adequately supported by the results?

Partly

Is the background of the case’s history and progression described in sufficient detail?

Partly

Reviewer Expertise:

urban morphology, AI, urban resilient, sustainable architecture, sustainable urbanism, building typology, schools, smart cities, urban regeneration,

I confirm that I have read this submission and believe that I have an appropriate level of expertise to confirm that it is of an acceptable scientific standard.

## References

[1] : Sustainable rural housing in cold climates: a model for Rumicruz-Ecuador. *F1000Research* .2025;14: 10.12688/f1000research.162591.2 10.12688/f1000research.162591.2 41312212 PMC12648017

# Reviewer report: Reviewer response for version 2

**DOI:** 10.5256/f1000research.185309.r430819

**Published:** 2025-11-25

**Authors:** Dakshayini R. Patil

**Affiliations:** 1 BMS College of Architecture, Bengaluru, Karnataka, India

The paper addresses an immediate concern in most countries of the world battling the diminishing vernacular architectural attributes. I have read the elaborate reviewer feedback and response from authors, taking care of the raised aspects. Hence, it is a well grown responsible manuscript at this stage. The sensitivity of the proposal, adoption of tools  and simulation-supported results are appreciated. 

 However, the article could have gained by improving overall sentence structuring and grammar improvement. Improvement in terms of better clarity, structuring. For eg. page no 4, third para, third sentence ending, same para last sentence having twice 'THUS'.... Observed several very small paragraphs across the script, may be structured better to tighten the narration and reader experience.  

 Figure no 2- Psychometric or should it read Psychrometric chart?

 Page no 17- Is it Ecotec or Ecotect? spelling error if at all.

 Following few questions:

 1)    To which exact period does “recent decades” and “progressive abandonment,” refer to?

 2)    Are the construction models or architectural imports that have replaced vernacular typologies detailed out?

 3)    Are two houses representative of the community or case at hand?

 4)    In terms of Data disclosure, was it as supplementary data? Because it says ‘No data are associated with this article’...how?

Is the case presented with sufficient detail to be useful for teaching or other practitioners?

Yes

Is the work clearly and accurately presented and does it cite the current literature?

Yes

If applicable, is the statistical analysis and its interpretation appropriate?

Yes

Are all the source data underlying the results available to ensure full reproducibility?

Yes

Are the conclusions drawn adequately supported by the results?

Yes

Is the background of the case’s history and progression described in sufficient detail?

Yes

Reviewer Expertise:

Architecture, Urban Design, Pedagogy, Inclusive designs, Sustainability

I confirm that I have read this submission and believe that I have an appropriate level of expertise to confirm that it is of an acceptable scientific standard.

# Reviewer report: Reviewer response for version 2

**DOI:** 10.5256/f1000research.185309.r401212

**Published:** 2025-08-25

**Authors:** Natividad Garcia-Troncoso

**Affiliations:** 1 Escuela Superior Politécnica del Litoral (ESPOL), Guayaquil, Ecuador

I generally agree with the adjustments made and the process can proceed. I recommend acceptance.

Is the case presented with sufficient detail to be useful for teaching or other practitioners?

Yes

Is the work clearly and accurately presented and does it cite the current literature?

No

If applicable, is the statistical analysis and its interpretation appropriate?

Yes

Are all the source data underlying the results available to ensure full reproducibility?

Yes

Are the conclusions drawn adequately supported by the results?

Yes

Is the background of the case’s history and progression described in sufficient detail?

Yes

Reviewer Expertise:

NA

I confirm that I have read this submission and believe that I have an appropriate level of expertise to confirm that it is of an acceptable scientific standard.

# Reviewer report: Reviewer response for version 1

**DOI:** 10.5256/f1000research.178816.r397888

**Published:** 2025-07-16

**Authors:** Natividad Garcia-Troncoso

**Affiliations:** 1 Escuela Superior Politécnica del Litoral (ESPOL), Guayaquil, Ecuador

The article presents a well-structured and culturally sensitive proposal for sustainable rural housing in Rumicruz, Ecuador. The integration of local materials and bioclimatic design significantly improves thermal comfort and preserves cultural identity. While the conclusions are supported by simulation results, the methodology requires clarification regarding data collection techniques and sample description. Addressing these aspects will enhance the study’s reproducibility and scientific robustness. Overall, the article is relevant, timely, and appropriate for indexing with minimal revisions.

Is the case presented with sufficient detail to be useful for teaching or other practitioners?

Yes

Is the work clearly and accurately presented and does it cite the current literature?

No

If applicable, is the statistical analysis and its interpretation appropriate?

Yes

Are all the source data underlying the results available to ensure full reproducibility?

Yes

Are the conclusions drawn adequately supported by the results?

Yes

Is the background of the case’s history and progression described in sufficient detail?

Yes

Reviewer Expertise:

Civil Engineering – Structural Design and AnalysisSustainable Construction and Materials

I confirm that I have read this submission and believe that I have an appropriate level of expertise to confirm that it is of an acceptable scientific standard.

# Reviewer report: Reviewer response for version 1

**DOI:** 10.5256/f1000research.178816.r380623

**Published:** 2025-05-27

**Authors:** Juan Andrés Sánchez García

**Affiliations:** 1 Universidad Veracruzada, Xalapa, Mexico

The introductory paragraph mentions:

 The indiscriminate use of modern materials has contributed to the loss of the diversity of traditional building styles and techniques, once a manifestation of local history.

 However, it does not address the types of materials, styles, and construction techniques used; this should be mentioned to provide context.

 It mentions that "it is essential to consider that this symbiosis manages bioclimatic, sustainable, and ecological strategies that should be rescued, as well as the traditional architectural forms, textures, and native colors, based on local materials and pigments that reflect the cultural identity of the region." In this case, sustainable and/or ecological bioclimatic strategies are mentioned as examples.

 The text states that "In this context, it is necessary to understand separately what rural housing refers to. Housing should be understood as a place that provides shelter, where the user experiences a sense of peace and appropriation of the place." However, it does not mention what housing conditions that respect the natural environment and reflect its culture have been.

 The text states: "However, in recent decades, the massive adoption of construction models alien to these contexts has generated a gap between traditional rural architecture and the demands of modern development." In this case, what period of time do the decades refer to and what are the construction models?

 It states that: "To this end, it is crucial to rescue and adapt traditional construction techniques to current demands." However, it does not mention which traditional construction techniques can be revived.

 It states that: "For this, local techniques and materials that avoid pollution and promote employment in the region are used." It does not mention which techniques and how employment is promoted.

 It is described as "This change also responds to a perceived need for modernization, which seeks to imitate foreign architectural styles without valuing the ancestral knowledge found in vernacular constructions." The foreign architectural style should be mentioned.

 It is stated that "The local situation increases when the number of existing Andean vernacular dwellings disappears over the years. Currently, in this study area, only six vernacular dwellings that present these outstanding cultural and architectural features are preserved." However, the time over time is not mentioned. To assess the decreasing change in the processes, it would be appropriate to mention the typologies of the six dwellings.

 2. In the methodology section, it states that "This study comprises qualitative and quantitative methods. In the qualitative section, a bibliographic exploration of guidelines related to" is required. However, qualitative research is not based on a bibliographical search of concepts. This section is called a theoretical framework or state of the art, so all research requires it. Qualitative methods in this section refer to instruments of a semiotic, phenomenological, ethnographic, etc. nature, and this research does not have that nature.

 In the case of quantitative methods, it states that "the quantitative approach evaluates the impact through a detailed diagnosis of the study site, offering effective solutions that respond to the real needs detected in the place where the proposal will be implemented." This is not found in the study. DOESN'T HAVE IT.

 It is mentioned that “The quantitative approach will be used for the collection of numerical data, which will provide accurate information on the spatial needs, as well as the functional and bioclimatic quality of the dwellings. In this way, it will be possible to develop a proposal that integrates design strategies in relation to the environment, architectural typology, materiality, construction, bioclimatic and efficiency in housing.” However, the methodology does not mention how the data was collected, nor do it mention the values ​​and instruments for spatial needs or environmental relationships.

 2.3. Research level

 This section mentions that "The exploratory level is focused on the can can can can can is the ...

 The phrase "is the can is the can" is often mentioned.

 However, the methodologies and results do not mention how the exploratory phase was carried out, which is important to mention to provide certainty about the results given the procedure.

 In section 2.4 Research Modality, it is mentioned that "The research adopts a dual approach, integrating inductive and analytical methods. The inductive approach focuses on observation and analysis of site conditions, allowing for the formulation of design assumptions tailored to site-specific needs."

 These are definitions; it is suggested that the inductive and analytical processes be specified and replaced by definitions.

 It is mentioned that: "Research procedures and techniques: A diagnosis of the current situation in the area will be made through field studies." However, the results do not mention the information on the available worldview and customs that made the proposal possible, i.e., the qualitative aspect.

 In the Study Population section, there is no mention of the homes diagnosed, what the representative sample was, or how the data was obtained; that is, was it a census or a multi-stage, representative, or systematic sampling process?

 3. Results

 It is mentioned that there were findings related to garbage accumulation, abandoned or half-built homes due to economic difficulties, soil degradation in areas and ravines, crop damage, and the introduction of materials that do not improve the thermal comfort of the inhabitants. The question is how this information was obtained or what technique was used to obtain this data.

 It is stated that: In urban-architectural terms, 95% of the houses are single-story, mostly built with block, tile, glass, brick, and reinforced concrete. Specifically, how many homes were studied?

 It is described that: The urban analysis also revealed that one-story rural dwellings are isolated, grow horizontally, and lack symmetry or continuity, surrounded by shrub and eucalyptus vegetation. The urban study should be specified since it was not mentioned in the methodology, as well as the technique used, if it was a spatial study.

 It is stated that: The vegetation at the study site is divided into four zones: moorland, natural forests, production zones, and abandoned areas and ravines, with the production zone being the most extensive.

 It would be appropriate to present a map determining the population of the homes and the vegetation areas studied.

 In section 3.2 Bioclimatic Requirements Analysis for Exteriors and Interiors, although a series of necessary data for this parameter is provided, it must be related to what was stated in the objective regarding comfort, so it must generate the way in which it affects or affects the Rumicruz home.

 The typology section adequately describes the housing attributes.

 The results mention that there are several housing studies, but they should mention which ones and their important attributes for comparison with the proposal in this document.

 It is mentioned that certain software was used, which should be included in the methodology and not in the conclusions.

 General Recommendations

 The history and evolution of the case should be described in terms of the social conditions and customs of the area to highlight the rarely mentioned ways of living.

 The conclusions need to return to the objectives and highlight that some parts were not met or were not presented as the previous diagnosis for use in other documentaries. The cultural loss that could be deciphered with what the authors refer to as a housing diagnosis was not addressed.

Is the case presented with sufficient detail to be useful for teaching or other practitioners?

Partly

Is the work clearly and accurately presented and does it cite the current literature?

Yes

If applicable, is the statistical analysis and its interpretation appropriate?

Not applicable

Are all the source data underlying the results available to ensure full reproducibility?

Yes

Are the conclusions drawn adequately supported by the results?

Partly

Is the background of the case’s history and progression described in sufficient detail?

Partly

Reviewer Expertise:

Architecture, design, and urban planning. Urban modeling, architectural design

I confirm that I have read this submission and believe that I have an appropriate level of expertise to confirm that it is of an acceptable scientific standard, however I have significant reservations, as outlined above.

# Response

**Published:** 2025-06-10

**Authors:** Karina Elizabeth Cajamarca Dacto

**Affiliations:** Faculty of Engineering, Universidad Nacional de Chimborazo, Riobamba, Chimborazo Province, Ecuador

I am sincerely grateful for the suggestions made to the article, which have contributed significantly to improving the quality of the research. In response to your comments, we have incorporated the aspects mentioned more precisely, thus strengthening the content of the study.

 Below are the sections that have been added to the document in response to your concerns. Likewise, the manuscript submitted to the journal has been updated, including the improvements based on the recommendations you kindly provided.

 Yours sincerely,

  

1. **The indiscriminate use indiscriminate use of modern materials has contributed to the loss of the diversity of traditional building styles and techniques, which were once a manifestation of local history. However, it does not address the types of materials, styles and construction techniques used; this should be mentioned to provide context.**

Likewise, according to the application of the field research, there is a marked contrast between the materials used in the current dwellings and in the vernacular dwellings of the area. The current houses have a predominance of concrete in the foundations, walls, structure and roof. They usually have two or more stories, are arranged contiguously and their openings are accessible. These constructions use materials acquired in the city and a mixed labor force, since personnel are often hired from the city.

 On the other hand, vernacular housing was mostly built with earth, wood, straw and reeds. It was built in isolation on the land, with a one-story typology, sloping roofs, and was built with local materials and labor.

**2. It mentions that “it is essential to consider that this symbiosis manages bioclimatic, sustainable and ecological strategies that must be rescued, as well as traditional architectural forms, textures and native colors, based on local materials and pigments that reflect the cultural identity of the region”. In this case, sustainable and/or ecological bioclimatic strategies are cited as examples.**

 On the other hand, some sustainable and/or ecological bioclimatic strategies that can be highlighted in the rural dwellings of Rumicruz focus on capturing solar gain to increase the interior temperature, due to the cold climate of the area. In vernacular dwellings, the use of openings was avoided to reduce heat loss as much as possible, and materials with high inertia and thermal conductivity were used, capable of capturing heat and transmitting it to the interior. An example of this is the use of earth, the predominant material in the walls of the vernacular dwellings studied. In addition, the longest sides of the house were oriented towards the east and west, in response to the sun's path in this geographical area.

**3. The text points out that “In this context, it is necessary to understand separately what rural housing refers to. Housing should be understood as a place that provides shelter, where the user experiences a sense of peace and appropriation of the place”. However, it does not mention which housing conditions have been respectful of the natural environment and reflect its culture.**

 Understanding that rural housing should be a way to improve the economy of the family, that is, it should be adapted to the conditions of the sector and the new realities, understanding the ways of life and socio-cultural constructions within a productive environment. (Pinto, 2009).

 Pinto,V.(2009). Rural housing in Ecuador: Challenges for sustainable and inclusive processes. Komuniacarte. Quito, Ecuador.p (3-20)

**4. The text states: “However, in recent decades, the massive adoption of construction models alien to these contexts has generated a gap between traditional rural architecture and the demands of modern development”. In this case, to what period of time do the decades refer and what are the construction models?**

 According to the detailed observation carried out in the community of Rumicruz, it has been possible to determine that vernacular housing has been losing continuity and being progressively replaced since the 1980s. That is to say, approximately four decades have passed since 1985, when houses with construction models foreign to rurality began to appear. These new buildings incorporate modern materials such as concrete, glass and steel in different elements, such as the structure, windows, doors and walls.

**5. He states that: “To this end, it is crucial to rescue and adapt traditional construction techniques to current demands”. However, he does not mention which traditional construction techniques can be revived.**

 The traditional construction techniques observed during the field research, and which could be rescued for application in the community's current housing, include the use of local materials such as earth, straw, wood and stone. These materials offer several benefits, including: the recovery of architectural identity, the use of resources with a smaller ecological footprint, better thermal properties and, consequently, greater thermal comfort inside the house.

**6. It states that: “This is done using local techniques and materials that avoid contamination and promote employment in the region”. It does not mention which techniques and how employment is promoted.**

 Through the implementation of the architectural proposal with materials from the area, its construction is promoted through local labor, which promotes the economic development of the community members and, at the same time, allows for the conservation and transmission of knowledge about this type of construction to future generations, by integrating them into the construction process.

 Within the proposed property, specific areas are defined for the cultivation and harvesting of local products such as potatoes, barley and corn. These products will later be stored in an infrastructure located on the site, designed for storage, allowing them to be marketed both in the locality and in the surrounding areas. This dynamic generates a network of economic sustenance for the users of the house and other members of the community. In addition, these products can be used for self-consumption, which contributes to guaranteeing the food sovereignty of its inhabitants.

**7. It is described as “This change also responds to a perceived need for modernization, which seeks to imitate foreign architectural styles without valuing the ancestral knowledge found in vernacular constructions”. It is worth mentioning the foreign architectural style.**

 Also, according to the observation of the architecture in the community of Rumicruz, the current dwellings present a modern architectural style, more linked to the urban than to the rural. This is evidenced by the use of concrete beam-column type structures, the presence of glass in the windows, accessible concrete slab roofs, as well as the use of steel in doors and in the protections of some windows.

**8. Through the observation of the community's architecture, it is possible to observe that in the construction of the community's houses, the local situation worsens when the number of existing Andean vernacular houses disappears over the years.**

 It is stated that “The local situation worsens when the number of existing Andean vernacular dwellings disappears over the years. Currently, in this study area, only six vernacular dwellings are preserved that present these outstanding cultural and architectural characteristics”. However, there is no mention of time over time. In order to evaluate the decreasing change in the processes, it would be opportune to mention the typologies of the six dwellings.

 Of the six Andean vernacular dwellings still preserved in the community, the oldest dates from 1949 and the most recent from 1960. All of these dwellings have a single-story rural typology, isolated on the property, without windows, with adobe load-bearing walls and sloping thatched and reed roofs. In other words, they are built with local materials and respond to local climatic conditions.

 However, from 1985 to the present, the houses in Rumicruz have undergone significant transformations in terms of their form, function, materials and typology, showing a progressive abandonment of their relationship with the environment and with the characteristics of rural housing.

**9. In the methodology section, it is stated that “This study comprises qualitative and quantitative methods. In the qualitative section, a bibliographic exploration of quality-related guidelines is required. However, qualitative research is not based on a literature search of concepts. This section is referred to as the theoretical framework or state of the art, so all research requires it. Qualitative methods in this section refer to instruments of a semiotic, phenomenological, ethnographic, etc. nature, and this research is not of that nature.**

In the case of quantitative methods, it states that “the quantitative approach evaluates the impact through a detailed diagnosis of the study site, offering effective solutions that respond to the real needs detected in the place where the proposal will be implemented”. This is not found in the study. IT DOES NOT. ************

 It is mentioned that “the quantitative approach will be used for the collection of numerical data, which will provide accurate information on the spatial needs, as well as the functional and bioclimatic quality of the dwellings. In this way, it will be possible to develop a proposal that integrates design strategies in relation to the environment, architectural typology, materiality, construction, bioclimatic and housing efficiency”. However, the methodology does not mention how the data was collected, nor does it mention values and instruments for spatial needs or environmental relationships.

 For data collection, the PDOT of the parish of Calpi was used as the main bibliographic source, which provides several useful inputs as a starting point, especially with regard to numerical data on population, economic activities and risk situations.

 Along with this information, a concise analysis of architectural references in Ecuadorian páramo areas, such as the community of Rumicruz, was proposed, highlighting strategies applied for the air conditioning of spaces, the materials used, their properties, the associated cultural identity and their environmental impact.

 In addition, the bibliography of Condor (2019), who conducted an analysis on the spaces and their relationship with the environment in the vernacular dwellings of Rumicruz, was reviewed.

 Based on this information, it was possible to develop an adequate field research, which allowed contrasting what was studied in the literature review and understanding the current situation of the place.

10. Level of research In this section it is mentioned that “The exploratory level focuses on the can can can can can is the ....The phrase “it's the can can, it's the can can” is often mentioned. However, the methodologies and results do not mention how the exploratory phase was carried out, which is important to mention in order to give certainty about the results given the procedure.***********

 The present research is situated at an exploratory and propositional level. The exploratory approach allows understanding the characteristics of the rural environment of the community of Rumicruz, province of Chimborazo, through the qualitative analysis of the vernacular architecture and the physical, cultural and climatic context. Through the bibliographic study, field observation and comparative analysis of two representative dwellings, one vernacular from 1989 and another contemporary from 2016, the architectural elements, materials, construction techniques and thermal comfort criteria that have influenced the configuration of housing in the sector were identified. This exploratory phase was essential to diagnose the problems affecting the community, both in terms of sustainability and loss of architectural identity.

 The propositional approach, on the other hand, is based on the findings obtained during the exploratory phase and aims to develop an architectural proposal for sustainable rural housing that responds to the real needs of the population. This proposal considers criteria of energy efficiency, use of local resources, adaptation to the natural environment and rescue of cultural values. It is based on bioclimatic design principles and on the analysis of quantitative data provided by sources such as the Development and Land Use Plan (PDOT) of the parish of Calpi, as well as on the results obtained from the media.

**11. In section 2.4 Research modality, it is mentioned that “The research adopts a dual approach, integrating inductive and analytical methods. The inductive approach focuses on observation and analysis of site conditions, allowing for the formulation of design assumptions tailored to site-specific needs.”**

These are definitions; it is suggested that the inductive and analytical processes be specified and replaced by definitions.*****************

 The research is developed through the application of two complementary methods: inductive and analytical, integrated in the methodological process for specific purposes.

 The inductive method was applied through direct observation of the study site during the field trips carried out in the community of Rumicruz. This observation made it possible to identify recurrent patterns and characteristics in the built environment, such as the use of local materials, the orientation strategies of the houses and the climatic conditions of the páramo. Based on these elements, preliminary guidelines were established that guided the development of the sustainable architectural proposal, adjusted to the real needs of the place.

 On the other hand, the analytical method was implemented through the review of specialized literature, the comparative analysis between a vernacular house built in 1989 and a contemporary house of 2016, and the use of Ecotec software to evaluate the thermal behavior of both typologies. This analysis made it possible to break down each architectural element and contextualize its influence on thermal comfort, spatial functionality and energy efficiency. The results obtained provided a solid technical basis to justify the design decisions adopted in the final proposal.

**12.** **It is mentioned that: “Research procedures and techniques: A diagnosis of the current situation of the area will be carried out through field studies”. However, the results do not mention the information on the available cosmovision and customs that made the proposal possible, that is, the qualitative aspect.**

 The research has a qualitative-quantitative approach. The qualitative component focuses on identifying the qualities of the sector through a bibliographic exploration of guidelines related to the relationship between architecture and environment, architectural typology, materiality and construction techniques, bioclimatic design principles and efficient housing criteria. This information provides a solid conceptual basis for the formulation of a sustainable proposal.

 An analysis of architectural references in Ecuadorian páramo areas, such as the community of Rumicruz, was also carried out, highlighting strategies applied for the air conditioning of spaces, the materials used, their properties, their environmental impact and the cultural identity associated with these elements. In addition, a comparison of two houses in Rumicruz, built in 1989 and 2016 respectively a vernacular house and a current house, was carried out, which allowed identifying key aspects related to their architectural identity, functionality, materials, construction systems and guidelines. These aspects were rescued and incorporated into the new proposal [20]. This analysis was performed using Ecotec software, obtaining relevant information on the influence of the use of local materials with better inertia and thermal conductivity, which favors a more comfortable interior temperature. It also highlighted the importance of the proper orientation resp

**13. The Study Population section does not mention which households were diagnosed, what the representative sample was or how the data were obtained, i.e., was it a census or a multi-statistical, representative or systematic sampling process?**

 It has been decided to conduct the research in the head of the community of Rumicruz. To this end, information will initially be gathered from the Development and Land Use Plan (PDOT) of the Calpi parish, complemented by a critical observation during the site visit. Subsequently, a diagnosis will be carried out to obtain specific data on the needs of the sector.

 In this process, a direct consultation was carried out with the Rumicruz community authorities, who have updated data from the last population census, which registered a total of 350 families. However, due to the temporary or permanent displacement of some members for reasons of study or work, it was considered pertinent to hold discussions with a representative of each family nucleus present in the community. As a result, a representative sample of 80 surveyed inhabitants was obtained.

**14. 3. Results**

**It is mentioned that there were findings related to garbage accumulation, abandoned or half-built houses due to economic difficulties, soil degradation in areas and ravines, crop damage, and the introduction of materials that do not improve the thermal comfort of the inhabitants. The question is how this information was obtained or what technique was used to obtain this data.**

 Following the suggested methodology, the analysis of the Rumicruz sector and based on the bibliographic information available in the Calpi Parish Land Use Plan and exploratory field research identified problems such as the accumulation of garbage, abandoned or half-built houses due to economic difficulties, soil degradation on land and streams, damage to crops and the introduction of materials that do not improve the comfort of the inhabitants. The community is linked to economic livelihood activities, which makes the house mainly a shelter for the night, while the corridor or doorway of the house is used as a social space for rest and conversation.

**15. It is stated that: In urban-architectural terms, 95% of the dwellings are single-story, mostly built with block, tile, glass, brick and reinforced concrete. Specifically, how many dwellings were studied?**

 It is described that: The urban analysis also revealed that rural one-story dwellings are isolated, grow horizontally and lack symmetry or continuity, surrounded by shrub and eucalyptus vegetation. The urban study should be specified since it was not mentioned in the methodology, as well as the technique used, if it was a spatial study.

 Likewise, the study was carried out on a total of 79 houses, that is, all the houses in the community. The research was carried out through the preparation of urban-architectural analysis sheets, dividing the community into five sections. In each section, a compendium of the characteristics observed in the corresponding houses was compiled, including aspects such as materiality, typology, vegetation, symmetry, continuity, among others.

 From the urban-architectural point of view, 95% of the houses are one-story, mostly built with block, tile, glass, brick and reinforced concrete. The roofs are flat and accessible, while the windows are small, limiting the entry of natural light and reflecting a lack of consistency in design between dwellings.

 The urban analysis also revealed that the one-story rural dwellings are isolated, with horizontal growth, lacking symmetry and continuity, and surrounded mainly by vegetation consisting of shrubs and eucalyptus trees.

**16. It is stated that: The vegetation of the study site is divided into four zones: paramo, natural forests, production zones and abandoned zones and ravines, with the production zone being the most extensive.**

 It would be convenient to present a map that determines the pPopulation of the dwellings and vegetation zones studied.

 The population of Rumicruz is located in the production zone because it covers the entire consolidated and built-up area.

**17. In section 3.2 Analysis of Bioclimatic Requirements for Exteriors and Interiors, although a series of necessary data is provided for this parameter, it should be related to the objective regarding comfort, so it should generate the way in which it affects or affects the Rumicruz house.**

*In this analysis, two graphs are presented: the bioclimatic chart and the psychrometric chart, which basically provide the guidelines for the ideal comfort level in exteriors and the second allows us to have an approach to the type of strategies that should be applied in the dwelling as such. In this way, the characteristics that the strategies proposed should have in order to have an ideal comfort level are defined in a general way, and with the bibliographic review of Deffis (1994) some examples are established. However, this affects the Rumicruz houses in a positive way because it provides a series of answers to the adjacent climatic conditions, sustainable solutions that can be applied according to the needs and creativity of the users..*

**18. The typology section adequately describes the housing attributes. The results mention that several housing studies exist, but mention should be made of which ones and their important attributes for comparison with the proposal in this document.**

 The analysis was carried out by comparing two houses in the community of Rumicruz, built in 1989 and 2016, respectively: a vernacular house and a current house. From this comparison, key aspects related to their architectural identity, functionality, materials, construction systems and guidelines were identified, which were rescued and incorporated into the new proposal [20].

 The study used Ecotec software, through which relevant information was obtained, highlighting the influence of the use of local materials with better inertia and thermal conductivity, which allow maintaining a more comfortable indoor temperature. Likewise, the importance of an adequate orientation of the house with respect to solar movement and prevailing winds was highlighted, which favors a better use of thermal gains towards the interior of the building.

 For these reasons, the 1989 vernacular house achieved a higher thermal comfort index compared to the house built in 2016.

19. It is mentioned that some software was used, which should be included in the methodology and not in the conclusions. ***********

**Recommendations** **general The history and evolution of the case should be described in terms of the social conditions and customs of the area to highlight the rarely mentioned ways of life.**

 The diagnosis carried out in the Rumicruz community indicates that approximately 60% of the families are made up of four members: father, mother and two children. Therefore, the proposal considers a family of four as the main users. According to the Plan de Desarrollo y Ordenamiento Territorial (PDOT) of Calpi (2019), the main economic activity is agriculture. The father is also a bricklayer, a job that many Rumicruz residents do in nearby cities such as Riobamba. The mother takes care of household chores and is also involved in agriculture, while the children, aged 14 and 12, attend school and help with agricultural work, a common practice in the rural area. The needs identified for the family include areas for planting crops on the property and outdoor and recreational spaces, as well as specific spaces for the children to study and do their homework, as they currently have to use common areas such as the dining room or living room. The daily workday begins very early, between 4 and 5 a.m., so it is necessary for the house to be adequately air-conditioned from early in the morning.

 Throughout the day, members devote most of their time to work, academic and domestic activities, leaving little time for leisure. Agricultural activities involve daily commutes of between 2 and 5 kilometers, often on foot, due to the lack of adequate areas for cultivation within their properties. This situation also affects the school performance of the children, who must help with the farm work and travel distances that take time away from their studies. Finally, the house is most occupied at night, from 5 p.m. to 5 a.m., so it is a priority to ensure efficient indoor climate control during these hours. To this end, we seek to maximize direct and indirect thermal gains during the day, taking advantage of solar radiation to heat the interior spaces.

# Response

**Published:** 2025-07-18

**Authors:** Karina Elizabeth Cajamarca Dacto

**Affiliations:** Faculty of Engineering, Universidad Nacional de Chimborazo, Riobamba, Chimborazo Province, Ecuador

I am sincerely grateful for the suggestions made to the article, which have contributed significantly to improving the quality of the research. In response to your comments, we have incorporated the aspects mentioned more precisely, thus strengthening the content of the study.

 Below are the sections that have been added to the document in response to your concerns. Likewise, the manuscript submitted to the journal has been updated, including the improvements based on the recommendations you kindly provided.

 Yours sincerely,

  

1. **The indiscriminate use indiscriminate use of modern materials has contributed to the loss of the diversity of traditional building styles and techniques, which were once a manifestation of local history. However, it does not address the types of materials, styles and construction techniques used; this should be mentioned to provide context.**

Likewise, according to the application of the field research, there is a marked contrast between the materials used in the current dwellings and in the vernacular dwellings of the area. The current houses have a predominance of concrete in the foundations, walls, structure and roof. They usually have two or more stories, are arranged contiguously and their openings are accessible. These constructions use materials acquired in the city and a mixed labor force, since personnel are often hired from the city.

 On the other hand, vernacular housing was mostly built with earth, wood, straw and reeds. It was built in isolation on the land, with a one-story typology, sloping roofs, and was built with local materials and labor.

**2. It mentions that “it is essential to consider that this symbiosis manages bioclimatic, sustainable and ecological strategies that must be rescued, as well as traditional architectural forms, textures and native colors, based on local materials and pigments that reflect the cultural identity of the region”. In this case, sustainable and/or ecological bioclimatic strategies are cited as examples.**

 On the other hand, some sustainable and/or ecological bioclimatic strategies that can be highlighted in the rural dwellings of Rumicruz focus on capturing solar gain to increase the interior temperature, due to the cold climate of the area. In vernacular dwellings, the use of openings was avoided to reduce heat loss as much as possible, and materials with high inertia and thermal conductivity were used, capable of capturing heat and transmitting it to the interior. An example of this is the use of earth, the predominant material in the walls of the vernacular dwellings studied. In addition, the longest sides of the house were oriented towards the east and west, in response to the sun's path in this geographical area.

**3. The text points out that “In this context, it is necessary to understand separately what rural housing refers to. Housing should be understood as a place that provides shelter, where the user experiences a sense of peace and appropriation of the place”. However, it does not mention which housing conditions have been respectful of the natural environment and reflect its culture.**

 Understanding that rural housing should be a way to improve the economy of the family, that is, it should be adapted to the conditions of the sector and the new realities, understanding the ways of life and socio-cultural constructions within a productive environment. (Pinto, 2009).

 Pinto,V.(2009). Rural housing in Ecuador: Challenges for sustainable and inclusive processes. Komuniacarte. Quito, Ecuador.p (3-20)

**4. The text states: “However, in recent decades, the massive adoption of construction models alien to these contexts has generated a gap between traditional rural architecture and the demands of modern development”. In this case, to what period of time do the decades refer and what are the construction models?**

 According to the detailed observation carried out in the community of Rumicruz, it has been possible to determine that vernacular housing has been losing continuity and being progressively replaced since the 1980s. That is to say, approximately four decades have passed since 1985, when houses with construction models foreign to rurality began to appear. These new buildings incorporate modern materials such as concrete, glass and steel in different elements, such as the structure, windows, doors and walls.

**5. He states that: “To this end, it is crucial to rescue and adapt traditional construction techniques to current demands”. However, he does not mention which traditional construction techniques can be revived.**

 The traditional construction techniques observed during the field research, and which could be rescued for application in the community's current housing, include the use of local materials such as earth, straw, wood and stone. These materials offer several benefits, including: the recovery of architectural identity, the use of resources with a smaller ecological footprint, better thermal properties and, consequently, greater thermal comfort inside the house.

**6. It states that: “This is done using local techniques and materials that avoid contamination and promote employment in the region”. It does not mention which techniques and how employment is promoted.**

 Through the implementation of the architectural proposal with materials from the area, its construction is promoted through local labor, which promotes the economic development of the community members and, at the same time, allows for the conservation and transmission of knowledge about this type of construction to future generations, by integrating them into the construction process.

 Within the proposed property, specific areas are defined for the cultivation and harvesting of local products such as potatoes, barley and corn. These products will later be stored in an infrastructure located on the site, designed for storage, allowing them to be marketed both in the locality and in the surrounding areas. This dynamic generates a network of economic sustenance for the users of the house and other members of the community. In addition, these products can be used for self-consumption, which contributes to guaranteeing the food sovereignty of its inhabitants.

**7. It is described as “This change also responds to a perceived need for modernization, which seeks to imitate foreign architectural styles without valuing the ancestral knowledge found in vernacular constructions”. It is worth mentioning the foreign architectural style.**

 Also, according to the observation of the architecture in the community of Rumicruz, the current dwellings present a modern architectural style, more linked to the urban than to the rural. This is evidenced by the use of concrete beam-column type structures, the presence of glass in the windows, accessible concrete slab roofs, as well as the use of steel in doors and in the protections of some windows.

**8. Through the observation of the community's architecture, it is possible to observe that in the construction of the community's houses, the local situation worsens when the number of existing Andean vernacular houses disappears over the years.**

 It is stated that “The local situation worsens when the number of existing Andean vernacular dwellings disappears over the years. Currently, in this study area, only six vernacular dwellings are preserved that present these outstanding cultural and architectural characteristics”. However, there is no mention of time over time. In order to evaluate the decreasing change in the processes, it would be opportune to mention the typologies of the six dwellings.

 Of the six Andean vernacular dwellings still preserved in the community, the oldest dates from 1949 and the most recent from 1960. All of these dwellings have a single-story rural typology, isolated on the property, without windows, with adobe load-bearing walls and sloping thatched and reed roofs. In other words, they are built with local materials and respond to local climatic conditions.

 However, from 1985 to the present, the houses in Rumicruz have undergone significant transformations in terms of their form, function, materials and typology, showing a progressive abandonment of their relationship with the environment and with the characteristics of rural housing.

**9. In the methodology section, it is stated that “This study comprises qualitative and quantitative methods. In the qualitative section, a bibliographic exploration of quality-related guidelines is required. However, qualitative research is not based on a literature search of concepts. This section is referred to as the theoretical framework or state of the art, so all research requires it. Qualitative methods in this section refer to instruments of a semiotic, phenomenological, ethnographic, etc. nature, and this research is not of that nature.**

In the case of quantitative methods, it states that “the quantitative approach evaluates the impact through a detailed diagnosis of the study site, offering effective solutions that respond to the real needs detected in the place where the proposal will be implemented”. This is not found in the study. IT DOES NOT. ************

 It is mentioned that “the quantitative approach will be used for the collection of numerical data, which will provide accurate information on the spatial needs, as well as the functional and bioclimatic quality of the dwellings. In this way, it will be possible to develop a proposal that integrates design strategies in relation to the environment, architectural typology, materiality, construction, bioclimatic and housing efficiency”. However, the methodology does not mention how the data was collected, nor does it mention values and instruments for spatial needs or environmental relationships.

 For data collection, the PDOT of the parish of Calpi was used as the main bibliographic source, which provides several useful inputs as a starting point, especially with regard to numerical data on population, economic activities and risk situations.

 Along with this information, a concise analysis of architectural references in Ecuadorian páramo areas, such as the community of Rumicruz, was proposed, highlighting strategies applied for the air conditioning of spaces, the materials used, their properties, the associated cultural identity and their environmental impact.

 In addition, the bibliography of Condor (2019), who conducted an analysis on the spaces and their relationship with the environment in the vernacular dwellings of Rumicruz, was reviewed.

 Based on this information, it was possible to develop an adequate field research, which allowed contrasting what was studied in the literature review and understanding the current situation of the place.

10. Level of research In this section it is mentioned that “The exploratory level focuses on the can can can can can is the ....The phrase “it's the can can, it's the can can” is often mentioned. However, the methodologies and results do not mention how the exploratory phase was carried out, which is important to mention in order to give certainty about the results given the procedure.***********

 The present research is situated at an exploratory and propositional level. The exploratory approach allows understanding the characteristics of the rural environment of the community of Rumicruz, province of Chimborazo, through the qualitative analysis of the vernacular architecture and the physical, cultural and climatic context. Through the bibliographic study, field observation and comparative analysis of two representative dwellings, one vernacular from 1989 and another contemporary from 2016, the architectural elements, materials, construction techniques and thermal comfort criteria that have influenced the configuration of housing in the sector were identified. This exploratory phase was essential to diagnose the problems affecting the community, both in terms of sustainability and loss of architectural identity.

 The propositional approach, on the other hand, is based on the findings obtained during the exploratory phase and aims to develop an architectural proposal for sustainable rural housing that responds to the real needs of the population. This proposal considers criteria of energy efficiency, use of local resources, adaptation to the natural environment and rescue of cultural values. It is based on bioclimatic design principles and on the analysis of quantitative data provided by sources such as the Development and Land Use Plan (PDOT) of the parish of Calpi, as well as on the results obtained from the media.

**11. In section 2.4 Research modality, it is mentioned that “The research adopts a dual approach, integrating inductive and analytical methods. The inductive approach focuses on observation and analysis of site conditions, allowing for the formulation of design assumptions tailored to site-specific needs.”**

These are definitions; it is suggested that the inductive and analytical processes be specified and replaced by definitions.*****************

 The research is developed through the application of two complementary methods: inductive and analytical, integrated in the methodological process for specific purposes.

 The inductive method was applied through direct observation of the study site during the field trips carried out in the community of Rumicruz. This observation made it possible to identify recurrent patterns and characteristics in the built environment, such as the use of local materials, the orientation strategies of the houses and the climatic conditions of the páramo. Based on these elements, preliminary guidelines were established that guided the development of the sustainable architectural proposal, adjusted to the real needs of the place.

 On the other hand, the analytical method was implemented through the review of specialized literature, the comparative analysis between a vernacular house built in 1989 and a contemporary house of 2016, and the use of Ecotec software to evaluate the thermal behavior of both typologies. This analysis made it possible to break down each architectural element and contextualize its influence on thermal comfort, spatial functionality and energy efficiency. The results obtained provided a solid technical basis to justify the design decisions adopted in the final proposal.

**12.** **It is mentioned that: “Research procedures and techniques: A diagnosis of the current situation of the area will be carried out through field studies”. However, the results do not mention the information on the available cosmovision and customs that made the proposal possible, that is, the qualitative aspect.**

 The research has a qualitative-quantitative approach. The qualitative component focuses on identifying the qualities of the sector through a bibliographic exploration of guidelines related to the relationship between architecture and environment, architectural typology, materiality and construction techniques, bioclimatic design principles and efficient housing criteria. This information provides a solid conceptual basis for the formulation of a sustainable proposal.

 An analysis of architectural references in Ecuadorian páramo areas, such as the community of Rumicruz, was also carried out, highlighting strategies applied for the air conditioning of spaces, the materials used, their properties, their environmental impact and the cultural identity associated with these elements. In addition, a comparison of two houses in Rumicruz, built in 1989 and 2016 respectively a vernacular house and a current house, was carried out, which allowed identifying key aspects related to their architectural identity, functionality, materials, construction systems and guidelines. These aspects were rescued and incorporated into the new proposal [20]. This analysis was performed using Ecotec software, obtaining relevant information on the influence of the use of local materials with better inertia and thermal conductivity, which favors a more comfortable interior temperature. It also highlighted the importance of the proper orientation resp

**13. The Study Population section does not mention which households were diagnosed, what the representative sample was or how the data were obtained, i.e., was it a census or a multi-statistical, representative or systematic sampling process?**

 It has been decided to conduct the research in the head of the community of Rumicruz. To this end, information will initially be gathered from the Development and Land Use Plan (PDOT) of the Calpi parish, complemented by a critical observation during the site visit. Subsequently, a diagnosis will be carried out to obtain specific data on the needs of the sector.

 In this process, a direct consultation was carried out with the Rumicruz community authorities, who have updated data from the last population census, which registered a total of 350 families. However, due to the temporary or permanent displacement of some members for reasons of study or work, it was considered pertinent to hold discussions with a representative of each family nucleus present in the community. As a result, a representative sample of 80 surveyed inhabitants was obtained.

**14. 3. Results**

**It is mentioned that there were findings related to garbage accumulation, abandoned or half-built houses due to economic difficulties, soil degradation in areas and ravines, crop damage, and the introduction of materials that do not improve the thermal comfort of the inhabitants. The question is how this information was obtained or what technique was used to obtain this data.**

 Following the suggested methodology, the analysis of the Rumicruz sector and based on the bibliographic information available in the Calpi Parish Land Use Plan and exploratory field research identified problems such as the accumulation of garbage, abandoned or half-built houses due to economic difficulties, soil degradation on land and streams, damage to crops and the introduction of materials that do not improve the comfort of the inhabitants. The community is linked to economic livelihood activities, which makes the house mainly a shelter for the night, while the corridor or doorway of the house is used as a social space for rest and conversation.

**15. It is stated that: In urban-architectural terms, 95% of the dwellings are single-story, mostly built with block, tile, glass, brick and reinforced concrete. Specifically, how many dwellings were studied?**

 It is described that: The urban analysis also revealed that rural one-story dwellings are isolated, grow horizontally and lack symmetry or continuity, surrounded by shrub and eucalyptus vegetation. The urban study should be specified since it was not mentioned in the methodology, as well as the technique used, if it was a spatial study.

 Likewise, the study was carried out on a total of 79 houses, that is, all the houses in the community. The research was carried out through the preparation of urban-architectural analysis sheets, dividing the community into five sections. In each section, a compendium of the characteristics observed in the corresponding houses was compiled, including aspects such as materiality, typology, vegetation, symmetry, continuity, among others.

 From the urban-architectural point of view, 95% of the houses are one-story, mostly built with block, tile, glass, brick and reinforced concrete. The roofs are flat and accessible, while the windows are small, limiting the entry of natural light and reflecting a lack of consistency in design between dwellings.

 The urban analysis also revealed that the one-story rural dwellings are isolated, with horizontal growth, lacking symmetry and continuity, and surrounded mainly by vegetation consisting of shrubs and eucalyptus trees.

**16. It is stated that: The vegetation of the study site is divided into four zones: paramo, natural forests, production zones and abandoned zones and ravines, with the production zone being the most extensive.**

 It would be convenient to present a map that determines the pPopulation of the dwellings and vegetation zones studied.

 The population of Rumicruz is located in the production zone because it covers the entire consolidated and built-up area.

**17. In section 3.2 Analysis of Bioclimatic Requirements for Exteriors and Interiors, although a series of necessary data is provided for this parameter, it should be related to the objective regarding comfort, so it should generate the way in which it affects or affects the Rumicruz house.**

*In this analysis, two graphs are presented: the bioclimatic chart and the psychrometric chart, which basically provide the guidelines for the ideal comfort level in exteriors and the second allows us to have an approach to the type of strategies that should be applied in the dwelling as such. In this way, the characteristics that the strategies proposed should have in order to have an ideal comfort level are defined in a general way, and with the bibliographic review of Deffis (1994) some examples are established. However, this affects the Rumicruz houses in a positive way because it provides a series of answers to the adjacent climatic conditions, sustainable solutions that can be applied according to the needs and creativity of the users..*

**18. The typology section adequately describes the housing attributes. The results mention that several housing studies exist, but mention should be made of which ones and their important attributes for comparison with the proposal in this document.**

 The analysis was carried out by comparing two houses in the community of Rumicruz, built in 1989 and 2016, respectively: a vernacular house and a current house. From this comparison, key aspects related to their architectural identity, functionality, materials, construction systems and guidelines were identified, which were rescued and incorporated into the new proposal [20].

 The study used Ecotec software, through which relevant information was obtained, highlighting the influence of the use of local materials with better inertia and thermal conductivity, which allow maintaining a more comfortable indoor temperature. Likewise, the importance of an adequate orientation of the house with respect to solar movement and prevailing winds was highlighted, which favors a better use of thermal gains towards the interior of the building.

 For these reasons, the 1989 vernacular house achieved a higher thermal comfort index compared to the house built in 2016.

19. It is mentioned that some software was used, which should be included in the methodology and not in the conclusions. ***********

**Recommendations** **general The history and evolution of the case should be described in terms of the social conditions and customs of the area to highlight the rarely mentioned ways of life.**

 The diagnosis carried out in the Rumicruz community indicates that approximately 60% of the families are made up of four members: father, mother and two children. Therefore, the proposal considers a family of four as the main users. According to the Plan de Desarrollo y Ordenamiento Territorial (PDOT) of Calpi (2019), the main economic activity is agriculture. The father is also a bricklayer, a job that many Rumicruz residents do in nearby cities such as Riobamba. The mother takes care of household chores and is also involved in agriculture, while the children, aged 14 and 12, attend school and help with agricultural work, a common practice in the rural area. The needs identified for the family include areas for planting crops on the property and outdoor and recreational spaces, as well as specific spaces for the children to study and do their homework, as they currently have to use common areas such as the dining room or living room. The daily workday begins very early, between 4 and 5 a.m., so it is necessary for the house to be adequately air-conditioned from early in the morning.

 Throughout the day, members devote most of their time to work, academic and domestic activities, leaving little time for leisure. Agricultural activities involve daily commutes of between 2 and 5 kilometers, often on foot, due to the lack of adequate areas for cultivation within their properties. This situation also affects the school performance of the children, who must help with the farm work and travel distances that take time away from their studies. Finally, the house is most occupied at night, from 5 p.m. to 5 a.m., so it is a priority to ensure efficient indoor climate control during these hours. To this end, we seek to maximize direct and indirect thermal gains during the day, taking advantage of solar radiation to heat the interior spaces.
